# Nanocarrier Strategies for Boron Drug Delivery in BNCT

**DOI:** 10.3390/mi17070846

**Published:** 2026-07-16

**Authors:** Sanjay Yadav, Efe Precious Onakpojeruo, Cedric Lansangan, Rameshwar Patil

**Affiliations:** 1 Department of Basic Sciences, Division of Cancer Science, School of Medicine, Loma Linda University, Loma Linda, CA 92350, USA; eonakpojeruo@students.llu.edu (E.P.O.); clansangan@students.llu.edu (C.L.); 2 Department of Neurosurgery, School of Medicine, Loma Linda University, Loma Linda, CA 92350, USA

**Keywords:** nanocarriers, boron neutron capture therapy, drug delivery, tumor targeting, high-LET radiation, tumor-selective radiation therapy, localized radiation, accelerator-based neutron source

## Abstract

Boron neutron capture therapy (BNCT) is a radiotherapeutic modality that enables tumor-targeted cell killing. The nuclear capture reaction between boron-10 (^10^B) and low-energy thermal neutrons produces high linear energy transfer (LET) particles (α-particles and recoiling lithium nuclei), each of which have short path lengths within the diameter of a single mammalian cell. The deposited energy creates clustered DNA double-strand breaks that are cytotoxic in these tumor cells while sparing the surrounding healthy tissues. This advantage makes BNCT a highly attractive treatment modality compared to conventional radiotherapy. Nevertheless, despite its theoretical precision, the clinical translation of BNCT remains constrained by suboptimal tumor-selective boron delivery; insufficient intracellular accumulation; and heterogeneous biodistribution profiles associated with conventional small-molecule-based boron agents, such as boronophenylalanine (BPA) and sodium borocaptate (BSH). While the development of new accelerator-based neutron sources (ABNSs) has renewed interest in BNCT, effective ^10^B delivery remains a major challenge. To address this, nanomedicine has been steadily on the rise in cancer research. In recent years, nanocarrier-based delivery systems have emerged as a transformative alternative delivery strategy. Nanodrugs offer several advantages over conventional small-molecule drugs, such as improved solubility, increased plasma half-life, enhanced permeability and retention in tumors, and active targeting, as well as decreased systemic toxicity and drug resistance. In recent years, nanocarrier-based delivery systems have emerged as a transformative strategy for ^10^B delivery. In this focused review, we will discuss various types of nanocarriers used for boron drug delivery that enhance boron loading efficiency and evaluate what enables their selective delivery to and accumulation within tumor cells.

## 1. Introduction

### 1.1. Overview of Boron Neutron Capture Therapy (BNCT)

Cancer remains one of the leading causes of mortality worldwide, necessitating the development of more precise and biologically targeted therapeutic strategies [1,2]. Among emerging modalities, BNCT has gained renewed attention as a binary, tumor-selective radiation therapy capable of delivering a highly localized cytotoxic radiation dose to cancer cells [1]. BNCT is dependent on the nuclear capture reaction between the stable isotope boron-10 (^10^B) and low-energy thermal neutrons. The reaction produces high linear energy transfer (LET) alpha (α)-particles and recoiling lithium-7 (^7^Li) nuclei (denoted as ^10^B(n,α)). These particles travel short path lengths (~5–9 µm), which is within the diameter of a single mammalian cell. The short distance confines lethal damage to boron-containing cells while sparing surrounding normal tissues [2]. Additionally, a therapeutic dose can be given in a single BNCT session compared to the fractionated doses used in photon radiotherapy. Conventional photon- and proton-based radiotherapy, meanwhile, rely on external beam irradiation and often result in significant radiotoxicity in healthy tissues due to nonspecific doses deposited around tumors. Recent advancements in accelerator-based neutron source (ABNS) development have revitalized clinical interest in BNCT by being eligible for installation inside hospitals, thus offering an in-house replacement of the off-site nuclear reactors currently being phased out [1,3]. Clinical studies performed outside of the USA within the last five years have demonstrated encouraging outcomes in recurrent head and neck cancers and glioblastomas, which highlights BNCT’s potential as a precision oncology modality [4,5].

However, despite its conceptual elegance and biological specificity, BNCT efficacy is fundamentally dependent on achieving sufficient and selective intracellular accumulation of ^10^B within tumor cells. The generally accepted therapeutic threshold (~20–30 µg ^10^B/g tumor tissue) and tumor-to-normal tissue ratio (>3–4:1) remain challenging to attain using conventional boron delivery agents. Consequently, selective, targeted boron delivery remains the central bottleneck in BNCT translation, limiting its widespread clinical adoption [6]. No boron delivery agents other than the first- and second-generation drugs (and their derivatives) have been accepted for clinical use.

### 1.2. Rationale and Scope of This Review

Nanocarriers have exploded in popularity in BNCT drug development research. These nanoscale(~1–100 nm in diameter) particles boast biocompatibility, capacity for functionalization, and magnetic properties, among other advantages. Many reviews on nanocarrier agents for BNCT have been published. Some have focused on the agents themselves, sometimes without in-depth consideration of why their properties are advantageous for certain tumor types but not for others. Given the rapid evolution of nanotechnology and its great potential in overcoming boron delivery limitations seen in BNCT, a focused synthesis of recent high-impact studies is necessary to provide clarity on current progress, future directions, and the following considerations:

- Advances in nanocarrier design for boron delivery;
- Comparative performance of different nanoplatforms;
- Strategies to enhance tumor targeting and intracellular delivery;
- Integration of BNCT with multimodal therapies;
- Translational challenges and clinical feasibility.

By consolidating recent innovations and critically evaluating nanocarrier-based approaches, this review aims to provide a comprehensive yet focused perspective on how nanotechnology can unlock the full therapeutic potential of BNCT in precision oncology. Importantly, compared to other publications, this review addresses the critical challenges of boron load and tumor targeting in next-generation delivery agents from chemistry- and biology-steeped points of view in terms of characteristics and other physicochemical properties ideal for boron delivery. This approach offers a unique vantage point from which to describe the literature assessed here in the context of ideal characteristics for ^10^B delivery into tumors.

### 1.3. Positioning of the Present Review Within the Existing Literature

Several comprehensive reviews have recently summarized advances in boron delivery systems and nanotechnology applications for boron neutron capture therapy (BNCT). These publications have substantially contributed to the understanding of nanocarrier development, boron chemistry, and emerging therapeutic strategies. Nevertheless, the rapid evolution of nanomedicine, together with recent progress in accelerator-based BNCT, multifunctional nanoplatforms, and biomimetic delivery systems, warrants an updated critical assessment of the field.

Unlike previous reviews, the present work was intentionally designed as a focused review rather than a broad narrative overview. Instead of comprehensively cataloging all available boron-containing nanomaterials, we critically evaluate approximately 89 recent high-impact studies, emphasizing the relationship between nanocarrier physicochemical properties and biological performance. Particular attention is devoted to how nanoparticle composition, particle size, surface charge, targeting ligands, intracellular trafficking, and boron loading influence therapeutic efficacy and clinical translation. Furthermore, whereas earlier reviews primarily describe individual nanocarrier systems, this review provides a comparative analysis across different nanoplatforms, including lipid-based, polymeric, dendrimeric, inorganic, carbon-based, and biomimetic carriers. These platforms are evaluated using common performance criteria, such as boron loading efficiency, tumor-targeting capability, biological barrier penetration, intracellular localization, therapeutic response following neutron irradiation, and translational readiness.

Another distinguishing feature of the present review is its emphasis on critical appraisal rather than descriptive summarization. Throughout the manuscript, representative studies are comparatively analyzed to identify the principal advantages, limitations, and remaining challenges associated with each nanocarrier platform.

Collectively, this focused review aims not only to summarize recent developments but also to identify the design principles most likely to facilitate successful clinical translation of next-generation boron delivery systems for BNCT. Table 1 visually organizes the manuscript’s unique contribution.

## 2. Fundamentals of BNCT

### 2.1. Physical and Nuclear Mechanisms of BNCT

Before diving directly into nanocarriers, a thorough exploration of BNCT is provided here. This two-step radiotherapeutic modality relies on a nuclear fission reaction that occurs at the cellular level [3]. After sufficient accumulation of a drug containing the stable isotope ^10^B within tumor cells and clearing from normal tissues and blood, the therapeutic effect is initiated when a ^10^B atom captures a low-energy (thermal) neutron. This neutron capture event produces an unstable form of boron-11 (^11^B), which undergoes immediate nuclear disintegration into high-energy charged particles according to the reaction [1]:

(1) B10+n→B*11→α (He4)+Li7+2.31 MeV

This reaction releases two primary particles—an α-particle (^4^He) and a recoiling lithium-7 (^7^Li) nucleus—along with kinetic energy (~2.31 MeV). Both particles are characterized by high linear energy transfer (LET), meaning they deposit a large amount of energy across a very short distance (termed as their path length). The path length of these particles in biological tissue is approximately 5–9 µm, which is comparable to the diameter of a mammalian cell [2].

The extremely short range of these high-LET particles is a defining feature of BNCT. It ensures that the destructive radiation effects are spatially confined to cells that have internalized ^10^B, thereby minimizing collateral damage to adjacent normal tissues. This microscopic selectivity distinguishes BNCT from conventional external beam radiotherapy, wherein dose deposition is less localized and often impacts healthy/normal tissues [10].

In addition to the primary reaction products, secondary gamma radiation may also be emitted; however, its contribution to biological damage is minimal compared to that of α and ^7^Li particles [11]. The overall therapeutic outcome of BNCT is therefore highly dependent on the microdistribution of boron at the cellular and subcellular levels, as well as the ability to deliver an adequate neutron flux (rate of neutrons transmitted per second at a specific surface area) to the tumor site. The overall BNCT dose comprises the following:

- The physical ^10^B dose in the form of ionizing energy deposited by high-LET α-particles and ^7^Li recoiling nuclei (^10^B(n,α)).
- The physical neutron dose from the protons released by the following:
  ○ The neutron capture reaction products from body tissue nitrogen atoms (^14^N(n,p) ^14^C).
  ○ Elastic collisions between fast/epithermal neutrons and the protons within body tissue hydrogen atoms (^1^H(n,n’)p), the latter of which have kinetic energy transferred to them from the neutrons and thus get “deflected” off of the hydrogen atoms.
- The photon dose from gamma (γ) rays released by the following:
  ○ The neutron beam from the beam port, which always contains some contaminating γ-rays.
  ○ Thermal neutron capture reaction by body tissue hydrogen atoms (^1^H(n,γ)^2^H).

**Figure 1:**
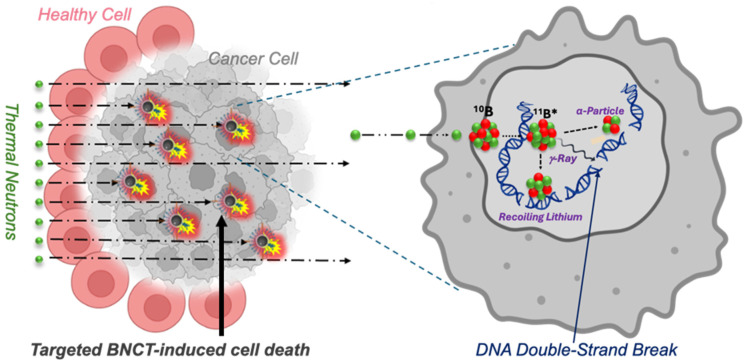
Schematic representation of BNCT principle. “^11^B*” refers to a highly-unstable form of ^11^B that disintegrates into high-LET particles. (Figure was created using Biorender.com, accessed in July 2026).

Recent technological advancements, particularly the development of accelerator-based neutron sources (ABNSs), have significantly improved the feasibility of BNCT by enabling compact, hospital-based neutron generation systems. These innovations have revitalized clinical interest in BNCT and expanded its applicability beyond that offered by reactor-based facilities. A schematic presentation of BNCT is shown in Figure 1.

### 2.2. Radiobiological Effects of BNCT

The radiobiological effectiveness of BNCT is primarily attributed to the high-LET radiation generated during the ^10^B(n,α) capture reaction. The high-LET particles released induce dense ionization tracks along their path lengths, leading to complex and clustered double-stranded DNA damage that is difficult for cancer cells to efficiently repair [10].

#### 2.2.1. High-LET DNA Damage

Unlike low-LET radiation (e.g., X-rays or gamma rays), which produces sparse and simple ionizations, high-LET radiation causes intense, localized energy deposition within nanometer-scale regions of DNA [12]. This results in:

- Multiple ionization events within a short spatial range;
- Complex DNA lesions involving closely spaced damage sites;
- Increased probability of DNA-damage-induced cytostaticity or cytotoxicity.

These characteristics significantly enhance the biological effectiveness of BNCT compared to conventional radiotherapy.

#### 2.2.2. DNA Double-Strand Breaks (DSBs)

Among the various forms of radiation-induced DNA damage, double-strand breaks (DSBs) are the most lethal. BNCT-induced high-LET particles generate [13]:

i. Direct DNA strand breakage through ionization of the DNA backbone.
ii. Indirect damage via radiolysis of the water that makes up the majority of human cell and body volume. This radiolysis also produces reactive oxygen species (ROS).

The clustered nature of DSBs induced by BNCT often overwhelms cellular repair pathways, such as non-homologous end joining (NHEJ) and homologous recombination (HR). As a result, incomplete repair leads to genomic instability and robust cell death.

##### 2.2.3. Cell Death Mechanisms

Accumulation of BNCT-induced complex and clustered DNA damage triggers multiple cell death pathways [14], including:

i. Apoptosis: Programmed cell death mediated by p53-independent activation of intrinsic mitochondrial-dependent pathways.
ii. Mitotic catastrophe: Failure of cells to properly complete mitosis due to chromosomal damage, leading to compounding genomic instability each following generation.
iii. Necrosis: Uncontrolled cell death resulting from severe damage to the cell membrane.
iv. Autophagy: Self-digestion processes activated under stress conditions.

Importantly, the high-LET nature of BNCT makes it particularly effective against radio-resistant tumors, including hypoxic cells and cancer stem-like cells, which are typically resistant to conventional low-LET radiation therapies.

Additionally, emerging evidence suggests that BNCT may stimulate immunogenic cell death, potentially enhancing antitumor immune responses through the release of damage-associated molecular patterns (DAMPs). This opens new avenues for combining BNCT with immunotherapy as co-adjuvants in future treatment strategies.

### 2.3. Clinical Requirements for Effective BNCT

Despite its strong theoretical and mechanistic advantages, the clinical success of BNCT is critically dependent on achieving specific biological and pharmacological conditions. These requirements ensure that a sufficient radiation dose is delivered selectively to tumor cells to induce cell death while minimizing toxicity to normal tissues.

A fundamental requirement for effective BNCT is the accumulation of an adequate amount of ^10^B within the tumor cells [15]. Clinical and preclinical studies have established that a minimum of 20 µg ^10^B per gram of tumor tissue is required to achieve robust therapeutic efficacy [16].

Achieving this concentration is challenging due to limitations in drug delivery, intratumor heterogeneity, and biological barriers. Insufficient boron accumulation leads to subtherapeutic radiation dose deposition, which highly reduces therapy efficacy.

#### 2.3.1. Tumor-to-Normal Tissue (T/N) Ratio

Equally important is the selectivity of boron accumulation, which is typically expressed as the tumor-to-normal tissue (T/N) ratio. For safe and effective BNCT, a ^10^B T/N ratio of ≥3–4:1 is generally required [17]. This ensures that radiation damage is preferentially localized within tumor tissues, thus minimizing the probability of healthy cell toxicity. Poor selectivity can result in unintended high-LET particle release in neutron-irradiated normal tissues surrounding the tumor, thus later leading to toxicity and limiting clinical applicability.

#### 2.3.2. Intracellular and Subcellular Localization

Beyond overall boron concentration, the intracellular distribution of ^10^B plays a crucial role in determining therapeutic outcome [18]. Ideally, ^10^B should be uniformly distributed within tumor cells, and preferentially localized near the cell nucleus to maximize DNA damage. When confined to extracellular spaces or poorly internalized within cells, ^10^B contributes minimally to therapeutic efficacy, as the short path length of high-LET particles limits their ability to reach the cell nucleus. Other important clinical requirements include:

I. Prolonged retention time of ^10^B in tumor tissue during neutron irradiation.
II. Rapid clearance from normal tissues and blood to reduce systemic toxicity.
III. Biocompatibility and low toxicity of boron delivery agents.
IV. Ability to cross biological barriers (e.g., the blood–brain barrier that separates the circulatory vasculature from the brain tissue, which has historically made drug development for BNCT of brain tumors extremely difficult—except for the success of BPA, which is currently administered for BNCT in Japan and other BNCT-using countries).

These stringent requirements highlight the limitations of conventional boron delivery agents. Thus, while ABNS technology has advanced to a point where effective neutron delivery is no longer a concern, there continues to be a need for novel and innovative delivery strategies, including via nanocarriers, to shuttle greater ^10^B content into tumors.

### 2.4. Physicochemical Determinants of Nanocarrier Performance in BNCT

Unlike conventional small-molecule boron compounds, nanocarrier-based delivery systems introduce numerous physicochemical variables that directly influence boron biodistribution and, consequently, the therapeutic efficacy BNCT. Although sufficient boron accumulation within tumor tissue is widely recognized as a prerequisite for successful treatment, growing evidence suggests that the biological effectiveness of BNCT depends not only on the amount of boron delivered but also on its spatial and intracellular distribution, both of which are strongly governed by nanocarrier design.

One of the most influential parameters is nanoparticle size. Particle size affects systemic circulation, vascular extravasation, tumor penetration, intracellular uptake, and clearance pathways. Nanoparticles ranging between approximately 50 and 150 nm generally demonstrate favorable pharmacokinetic behavior by avoiding rapid renal filtration while maintaining efficient accumulation through the enhanced permeability and retention (EPR) effect. Conversely, particles exceeding sizes of approximately 200 nm often exhibit reduced tumor penetration and increased sequestration by the reticuloendothelial system (RES), particularly within the liver and spleen. Extremely small nanoparticles (<10 nm), although capable of deeper tissue penetration, are rapidly eliminated through renal clearance, thereby limiting boron retention within tumors.

Surface characteristics further influence biological performance. Surface charge, commonly represented by the zeta potential, plays a central role in nanoparticle stability, protein adsorption, and cellular interactions. Positively charged nanoparticles generally exhibit enhanced cellular uptake due to electrostatic interactions with negatively charged cell membranes; however, an excessive positive charge may promote nonspecific protein adsorption, rapid macrophage uptake, and increased systemic toxicity. In contrast, neutral or slightly negative nanoparticles typically display improved colloidal stability, prolonged circulation times, and reduced protein corona formation, thereby preserving active targeting capability.

Surface functionalization with targeting ligands has become one of the most effective strategies for improving intracellular boron delivery. Ligands, including antibodies, peptides, aptamers, folate, transferrin, and amino acid analogs, facilitate receptor-mediated endocytosis, allowing boron-containing nanoparticles to accumulate selectively within tumor cells expressing the corresponding receptors. Such active targeting not only increases tumor boron concentration but also improves intracellular localization, an important determinant of BNCT efficacy given the extremely short path length of alpha particles and lithium nuclei generated during neutron capture.

Another important consideration is the subcellular localization of boron. Because alpha particles travel only a few micrometers, boron localized near the nucleus or within the cytoplasm produces significantly greater DNA damage than boron remaining in extracellular spaces or confined to vascular compartments. Consequently, nanocarriers capable of promoting endosomal escape and efficient intracellular trafficking are generally associated with improved therapeutic outcomes.

Collectively, these physicochemical characteristics—including particle size, morphology, surface chemistry, zeta potential, ligand density, and biodegradability—determine the pharmacokinetic and biological behavior of nanocarriers and should therefore be considered fundamental design parameters in the development of next-generation boron delivery systems.

### 2.5. BNCT Dose Components and Biological Dose Calculation

The therapeutic dose delivered during BNCT is fundamentally different from that of conventional external beam radiotherapy because it arises from the combined contribution of several radiation components. The total absorbed dose consists of:

- A boron dose, generated through the ^10^B(n,α)^7^Li reaction;
- A nitrogen dose, arising from the ^14^N(n,p)^14^C reaction;
- A hydrogen recoil dose, produced by fast neutron interactions;
- An accompanying gamma-ray dose.

Because these radiation components possess different biological effectiveness, the clinically relevant quantity is the biologically weighted dose, which incorporates both the relative biological effectiveness (RBE) and compound biological effectiveness (CBE) factors.

The biologically weighted BNCT dose can be expressed as:

$$D_{BW}=CBE\times D_{B}+RBE_{N}\times D_{N}+RBE_{H}\times D_{H}+D_{\gamma }$$

where $D_{B}$, $D_{N}$, $D_{H}$, and $D_{\gamma }$ represent the absorbed doses contributed by boron, nitrogen, hydrogen, and gamma radiation, respectively.

Unlike conventional radiotherapy, the boron dose component is directly influenced by nanoparticle-mediated boron biodistribution. Consequently, nanocarrier design becomes an important determinant of the biologically weighted dose delivered to tumor tissue. Improved intracellular boron localization increases the probability that high-LET alpha particles will traverse the cell nucleus, thereby maximizing DNA double-strand break formation while minimizing irradiation of adjacent healthy cells. Therefore, optimization of nanocarrier physicochemical properties directly contributes to improving both dosimetric accuracy and therapeutic efficacy.

## 3. Challenges in Boron Delivery

### 3.1. Biological Barriers

A survey into the reasons underlying ^10^B delivery problems for some tumors is needed. The clinical success of BNCT is fundamentally constrained by tumor-dependent penetrability of complex biological barriers. These barriers limit efficient delivery of boron agents into tumor tissues and also hinder uniform distribution of said agents in them. Among these, the blood–brain barrier (BBB) represents one of the most formidable obstacles, particularly in the treatment of central nervous system malignancies such as glioblastoma and diffuse midline glioma [19,20]. The BBB is a highly selective and tightly regulated interface comprising endothelial cells interconnected by tight junctions, supported by pericytes and astrocytic end-feet. This structural and functional complexity severely restricts the passive diffusion of most therapeutic agents and boron compounds, thereby limiting their ability to reach intracranial tumor sites at therapeutically relevant concentrations [19,20]. Although pathological conditions, such as tumor-induced angiogenesis, may partially disrupt BBB integrity, this disruption is often heterogeneous and insufficient for uniform drug distribution across the tumor mass.

In addition to the BBB, tumor heterogeneity poses a significant challenge to effective boron delivery. Solid tumors are characterized by spatial and temporal variability in vascularization, cellular density, metabolic activity, and receptor expression. This heterogeneity results in uneven distribution of boron agents, which leads to regions within the tumor that receive subtherapeutic boron concentrations. Consequently, even when average intratumor boron levels meet therapeutic thresholds, local pockets of subtherapeutic doses may allow tumor cells to survive and mediate recurrence. Furthermore, the abnormal architecture of tumor vasculature (often disorganized, leaky, and poorly perfused) contributes to boron agents having limited and inconsistent penetration into tumors. Elevated interstitial fluid pressure within tumors further impedes the extravasation and diffusion of therapeutic compounds, thus reducing their intratumoral penetration [21,22].

### 3.2. Pharmacokinetic Limitations

Beyond biological barriers, the pharmacokinetic properties of conventional boron delivery agents significantly limit their clinical effectiveness. A major challenge is the rapid systemic clearance of boron compounds from circulation, which reduces the probability of drug uptake by tumors prior to neutron irradiation [23,24]. Many boron agents exhibit short plasma half-lives due to renal clearance and hepatic trapping, thus resulting in poor accumulation within tumor tissues. This rapid elimination not only diminishes therapeutic efficacy, but also complicates determining optimal timing of neutron exposure, which must be precisely synchronized with both peak intratumor ^10^B concentration and minimal ^10^B concentration in normal tissues or blood.

Another critical issue is the low retention of boron within tumor cells. Even when initial uptake is achieved, boron compounds may not be retained long enough to still be present immediately prior to neutron irradiation, and thus this may lead to reduced physical ^10^B dose delivery at the cellular level. This limitation is exacerbated by the dynamic nature of tumor microenvironments, where continuous blood flow and metabolic activity can facilitate the washout of boron agents. Additionally, conventional boron compounds often exhibit nonspecific biodistribution, accumulating in normal tissues such as the liver, kidneys, and blood circulation as well as in the tumor. A specific example, 4-borono-L-phenylalanine, which will be described in Section 4, is preferentially taken up by tumors, but nonetheless has some accumulation in normal tissues. This lack of selectivity reduces the tumor-to-normal tissue ratio and thus increases the risk of off-target radiation damage following neutron exposure, thereby limiting the therapeutic index of BNCT [10].

### 3.3. Cellular-Level Challenges

At the cellular level, the effectiveness of BNCT is highly dependent not only on the presence of boron within tumor tissues but also on its precise intracellular localization. One of the key challenges is the limited ability of boron compounds to achieve efficient intracellular and nuclear delivery. Given the short path lengths of the high-LET particles generated by the ^10^B(n,α) reaction, boron must be localized near critical cellular targets as close to the nucleus as possible to maximize the cytotoxic effects [2]. However, many conventional boron delivery agents remain confined to extracellular spaces or are inadequately internalized by tumor cells, thereby significantly reducing their therapeutic potential.

Furthermore, even when boron compounds are internalized, cellular efflux mechanisms can actively reduce intracellular boron concentrations. Transport proteins and membrane pumps, such as ATP-binding cassette (ABC) transporters, can expel therapeutic agents from cells, and thus can contribute to decreased intracellular retention and resistance to treatment. These efflux processes are often upregulated in cancer cells, particularly in aggressive and drug-resistant tumors, which further complicates effective boron delivery. Collectively, these intracellular challenges highlight the importance of developing advanced delivery systems capable of facilitating efficient cellular uptake, enhancing nuclear localization, and overcoming or minimizing efflux-mediated loss [10,25].

## 4. Limitations of Conventional Boron Delivery Agents

To date, only two boron-containing compounds have been used in clinical BNCT protocols: 4-borono-L-phenylalanine (BPA) and sodium borocaptate (BSH) [26]. A landmark advancement in the clinical translation of BNCT was the regulatory approval of Steboronine^®^ (borofalan (^10^B)), a pharmaceutical formulation of BPA, by the Japanese Ministry of Health, Labour and Welfare in 2020 for the treatment of unresectable locally advanced or locally recurrent head and neck cancer [27]. Steboronine^®^ became the first clinically approved boron-containing drug specifically developed for BNCT, representing a major milestone in the transition of BNCT from an experimental treatment modality to an established clinical therapeutic option [27,28]. While BPA (Steboronine) exploits amino acid transport systems (particularly the L-type amino acid transporter 1, or LAT1) for tumor uptake, and BSH relies on passive diffusion mechanisms, both agents have been reported to suffer from critical limitations [26,29,30], including:

I. Low tumor selectivity and heterogeneous distribution.
II. Insufficient intracellular localization, particularly within nuclei.
III. Rapid systemic clearance and poor retention.
IV. Inability to effectively cross biological barriers, especially the BBB.

These limitations are particularly problematic in aggressive and infiltrative malignancies, such as glioblastoma and diffuse midline glioma. Achieving homogeneous boron distribution across the entirety of these tumors is essential. Moreover, tumor microenvironment heterogeneity (i.e., due to hypoxia, abnormal vasculature, and variable transporter expression) further exacerbates delivery inefficiencies. As highlighted in recent translational studies, inadequate boron accumulation remains a primary cause of suboptimal therapeutic outcomes and inconsistent clinical responses [31]. Therefore, the development of advanced delivery systems capable of enhancing boron payload, targeting specificity, and intracellular delivery is critical for the future success of BNCT [3].

### Emergence of Nanocarrier-Based Boron Delivery

Nanomedicine has emerged as a transformative approach in cancer research by offering significant advantages over conventional small-molecule drugs, including [14]:

i. Enhanced permeability and retention (EPR)-mediated tumor accumulation.
ii. Prolonged systemic circulation and improved pharmacokinetics.
iii. High drug loading capacity due to large surface area.
iv. Capability for surface functionalization with targeting ligand.
v. Controlled and stimuli-responsive drug release.
vi. Reduced systemic toxicity and improved therapeutic index.

As discussed in [32], nanoparticle-based nanomedicines can be broadly categorized into polymeric, lipid-based, and inorganic systems, each offering unique physicochemical and biological properties suitable for biomedical applications. These properties (particularly size, surface charge, shape, and modes of functionalization) critically influence biodistribution, cellular uptake, and therapeutic efficacy.

In the context of BNCT, nanocarriers provide a promising platform to overcome the intrinsic limitations of conventional boron agents [17] by enabling:

i. High boron loading capacity (multiple boron clusters per nanoparticle).
ii. Targeted delivery via ligands (antibodies, peptides, aptamers).
iii. Improved intracellular trafficking and nuclear localization.
iv. Multifunctionality (theranostic, combination therapy).

Recent studies, particularly those published in the last five years, have demonstrated the potential of nanocarriers to significantly enhance boron accumulation in tumor tissues while improving T/N. The remainder of this review will focus on describing the different classes nanocarriers, as well as summarizing and critically evaluating the findings of preclinical non-irradiation pharmacokinetic and neutron-irradiated BNCT studies.

## 5. Nanocarrier Strategies for Boron Delivery

**Figure 2:**
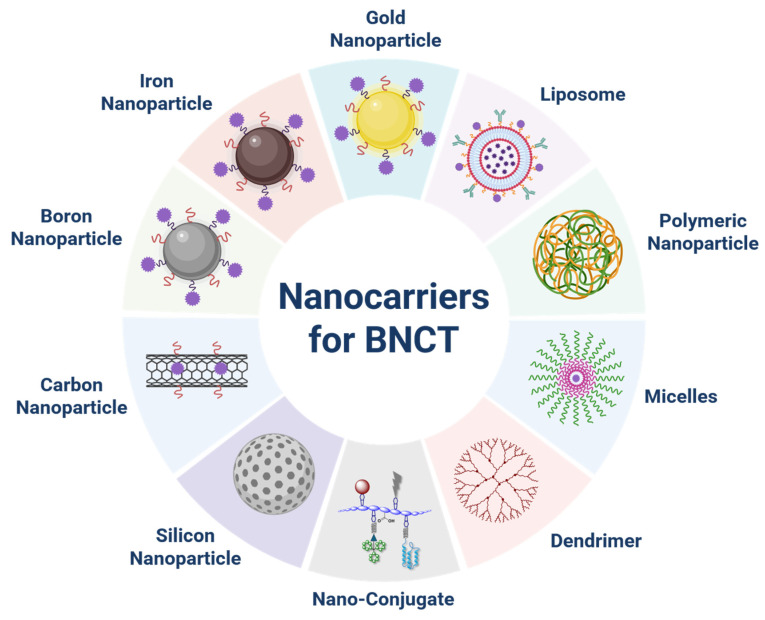
Major nanocarrier platforms for BNCT drug delivery. (Figure was created using Biorender.com accessed in July 2026).

The limitations associated with conventional boron delivery agents have driven the rapid development of nanocarrier-based systems (Figure 2) designed to accomplish the following: enhance tumor-selective accumulation, improve pharmacokinetics, and facilitate efficient intracellular delivery of boron-10.

Nanocarriers offer a versatile platform capable of integrating the following on a single carrier molecule: high boron payloads, targeting ligands, imaging agents, and stimuli-responsive functionalities [33]. Their tunable physicochemical properties (including size, surface charge, and morphology) enable improved interaction with biological systems, thereby overcoming many of the barriers discussed in the previous section. Nanocarriers improve medication delivery across the BBB by using natural transport mechanisms and tumor-induced BBB disruption. Similar principles allow penetration through other biological barriers as described earlier. Their diminutive dimensions; surface alterations; and targeted ligands, like angiopep-2, transferrin, or antibodies, facilitate receptor-mediated transcytosis and targeted intratumor accumulation.

Over the past five years, a wide range of nanocarrier systems have been investigated for BNCT applications, each offering distinct advantages and facing unique translational challenges. This section provides a detailed evaluation of the major classes of nanocarriers explored thus far for boron delivery [33].

### 5.1. Gold Nanoparticles

**Figure 3:**
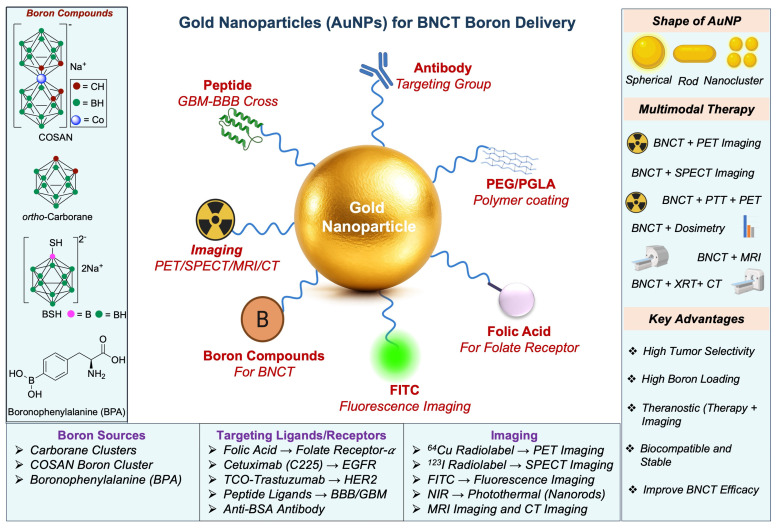
Gold nanoparticle platforms for boron delivery and multimodal applications. (Figure was created using Biorender and Figurelab AI (pro version)).

Multifunctional gold nanoparticles (AuNPs) have garnered much interest because of their desirable properties (Figure 3). Their inorganic metallic gold cores, encircled by monolayers of organic and/or biomolecules, are easily customized in terms of their size, shape, structure, and composition. Functionalization of AuNP surfaces with desirable ligands is achieved by most papers via thiolate bonds between surface gold atoms and thiol-containing molecules. The potential for utilizing AuNPs as boron carriers for BNCT has been the subject of several investigations.

In 2011, Bakeine and coworkers synthesized novel multifunctional nanovectors for targeted fluorescently trackable boron delivery. The group reported multilayer polyelectrolyte shells on AuNPs, each functionalized with fluorescein isothiocyanate (FITC), BPA, and folic acid [34]. In 2013, Wang et al. reported star-shaped PEGylated AuNPs conjugated with carborane clusters through click chemistry [35]. 2017 saw Wang and coworkers publish work on conjugating fluorescent gold nanoclusters (AuNCs) with boron-rich carborane derivatives to create a multifunctional nanoplatform for targeted cancer cell imaging. The ultra-small AuNCs displayed strong fluorescence, excellent biocompatibility, and efficient cellular uptake [36]. Later, in 2019, Llop and colleagues reported AuNPs loaded with boron capable of radiolabeling for BNCT. Their AuNP has potential as a multifunctional theranostic agent for BNCT, given its robust tumor accumulation and significant growth suppression with no reported toxicity following neutron irradiation in animal tumor models [37]. Also in 2019, Llop and coworkers published on boron-loaded radiolabeled AuNPs. This system allows effective tumor targeting, real-time in vivo tracking, and improved boron uptake. In a mouse tumor model, the nanoparticle exhibits significant tumor growth inhibition after neutron irradiation [37]. In that same year, Wu et al. demonstrated antibody-modified boron-containing AuNPs (123I-61-B-AuNPs) that allow for noninvasive imaging and targeted boron administration. This nanoparticle showed considerable tumor accumulation in microPET imaging and biodistribution investigation [38]. The Llop group reported in 2020 spherical AuNPs functionalized with tetrazine and loaded with the boron cluster COSAN, enabling the click reaction with TCO–trastuzumab and was thus suitable for the pre-targeting BNCT method. The system demonstrated stable, biocompatible multifunctionalization in vitro after being effectively coupled with TCO–trastuzumab. In vivo tracking was made possible via an alloyed gold core tagged with ^64^Cu, and a successful biorthogonal click reaction between the antibody and nanoparticles was verified [39]. In 2021, Llop and coworkers demonstrated multifunctional gold nanorods loaded with boron cluster COSAN agents efficiently accumulate at tumor sites and allow combined photothermal therapy and BNCT. Upon NIR irradiation and neutron exposure, a robust synergistic effect observed, resulting in substantial tumor growth inhibition with minimal systemic toxicity [40]. Later in the same year, Zaboronok and coworkers demonstrated the use of AuNPs as multifunctional agents in BNCT, allowing for both improved tumor dose deposition and in situ absorbed dose evaluation. By correlating nanoparticle distribution with neutron irradiation effects, the system allows improved dosimetry accuracy while increasing therapeutic efficacy against malignant tumors [41]. Peng and coworkers reported in 2021 developing MRI-detectable boron-containing AuNPs encapsulated with biodegradable PGLA polymer for BNCT. This system combined MRI and boron delivery, allowing real-time tracking of tumor accumulation [42]. In 2023, Potseleev et al. demonstrated a biodegradable polylactide-based nanocomposite incorporating gold complex compounds for absorbed dose diagnostics in BNCT. This technique allows in situ monitoring of the dispersion of neutron doses while preserving the material’s tumor location [43]. Amendola and coworkers also reported in 2023 their multifunctional Au-B theranostic nanovector, which was used for BNCT and X-ray radiotherapy, as well as with CT imaging, enabling localization and quantification of radiosensitizer in tissues. They demonstrated the synthesis of nanoparticles by laser ablation in liquid and stabilized with a biocompatible coating, such as dextran (DEX) or thiolated PEG [44]. In 2024, Zhang et al. reported peptide-functionalized AuNPs as a targeted boron carrier for BNCT in glioblastoma (GBM). Peptide ligands increase boron accumulation in tumor cells by improving transport across biological barriers. In vivo studies reveal significant tumor growth inhibition [45].

AuNP biosafety is highly dependent on both the composition of their surface ligands and their size. In 2021, Pulagam and colleagues reported AuNP nanorods multifunctionalized with PEG, boron-rich COSAN, and positron-emitting copper-64 [40]. (In this study, the authors found that cell viability was not significantly reduced in nanorod-treated gastric cancer cells nor in dermal fibroblasts, regardless of whether treatment was 24, 48, or 72 h. It was not reported whether tumor-bearing mice were monitored post-injection for weight changes (although for this study, a subcutaneous flank gastric tumor model was used). Testing the photothermal therapy component of the AuNPs indicated significant reduction in viability of spheroids of both types of cell lines when treated with AuNP + near-infrared irradiation. Interestingly, the group also carried out in vitro BNCT efficacy neutron irradiation studies (without replacing media containing AuNPs as in the PTT protocol) using the same cell lines and reported no significant decreases in viability/survival with AuNP alone. They also found that neutrons alone did significantly reduce viability, and that neutron irradiation synergistically increased this reduction significantly. Zhang and colleagues’ study from 2024 used ~34–46 nm AuNPs functionalized with either BSH+PEG or BSH+PEG-cRGD (cyclic RGD peptide) [45]. Only the latter AuNP had a near-neutral zeta potential, which is advantageous to avoid nonspecific binding while facilitating BBB binding. Their study found no significant differences in cell viability across AuNP-treated murine-derived cell lines of GBM, endothelia, and microglia, and no differences in viability were observed for cRGD-modified AuNPs as well. However, it is important to note that their animal model was of subcutaneous GBM rather than an orthotopic one, which completely ignores the major challenges posed by the BBB. Thus, despite their biosafety and very high ^10^B T/N, the authors’ final cRGD-PEG + BSH AuNPs will be difficult to translate to clinics without resorting to invasive craniotomy or cerebrospinal-fluid-mediated delivery through the BBB.

#### Theranostic Advantages of Gold Nanoparticles in BNCT

Beyond serving as efficient boron delivery vehicles, AuNPs possess unique physicochemical properties that distinguish them from most other nanocarrier systems and make them particularly attractive for theranostic applications in BNCT. Their high atomic number (Z = 79), excellent surface chemistry, chemical stability, and favorable biocompatibility enable AuNPs to function not only as drug carriers but also as multifunctional platforms capable of enhancing radiation response, facilitating molecular imaging, and supporting treatment verification. One of the principal advantages of AuNPs is their potential to enhance local radiation dose deposition. Although BNCT primarily derives its therapeutic effect from the high linear energy transfer (LET) α-particles and ^7^Li nuclei generated through the ^10^B(n,α)^7^Li reaction, the incorporation of gold into multifunctional nanoplatforms may further increase local energy deposition through secondary electron production following photon or mixed-field irradiation. This radiosensitizing capability has been extensively investigated in conventional radiotherapy and is increasingly being explored in combination with BNCT to maximize tumor cell killing while maintaining normal tissue sparing. Consequently, AuNPs provide an attractive strategy for integrating boron delivery with radiation dose enhancement, particularly in multimodal treatment protocols involving accelerator-based neutron sources. Another distinctive feature of AuNPs is their potential application in activation dosimetry. During neutron irradiation, naturally occurring ^197^Au readily undergoes neutron capture to produce radioactive ^198^Au through the following reaction:

Au197+n→Au198

Because the induced radioactivity of ^198^Au can be accurately quantified, gold has long been used as a neutron flux monitor in reactor physics and BNCT dosimetry. Incorporating AuNPs into boron delivery platforms therefore offers a unique opportunity to combine therapeutic boron transport with in situ neutron flux verification, potentially improving treatment planning and post-irradiation dose assessment. Such multifunctionality may contribute to more accurate estimation of the biologically effective dose delivered during BNCT and facilitate quality assurance in future clinical applications. In addition to their dosimetric advantages, AuNPs possess excellent computed tomography (CT) contrast properties owing to the high X-ray attenuation coefficient of gold. When combined with boron-containing ligands or gadolinium-based imaging agents, AuNPs can simultaneously support CT, magnetic resonance imaging (MRI), and optical imaging, enabling real-time visualization of nanoparticle biodistribution before neutron irradiation. This capability allows clinicians to verify tumor accumulation, optimize irradiation timing, and monitor treatment response, thereby supporting the growing field of image-guided BNCT. Surface modification further enhances the versatility of AuNPs. Their large surface-area-to-volume ratio facilitates conjugation with boron clusters, polyethylene glycol (PEG), antibodies, peptides, aptamers, and other targeting ligands, enabling active targeting of tumor-specific receptors while reducing nonspecific uptake by the reticuloendothelial system. Such multifunctional surface engineering allows AuNPs to simultaneously integrate targeted boron delivery, molecular imaging, radiation enhancement, and dosimetric monitoring within a single nanoplatform.

Despite these advantages, several challenges continue to limit the clinical translation of AuNP-based BNCT systems (Table 2). Gold is not biodegradable, and long-term accumulation within the liver, spleen, and other organs remains an important biosafety concern. Moreover, nanoparticle size, surface chemistry, and protein corona formation substantially influence biodistribution and clearance. Consequently, future studies should focus on optimizing AuNP physicochemical properties while balancing therapeutic efficacy, imaging capability, radiation enhancement, and long-term safety.

### 5.2. Iron Nanoparticles

**Figure 4:**
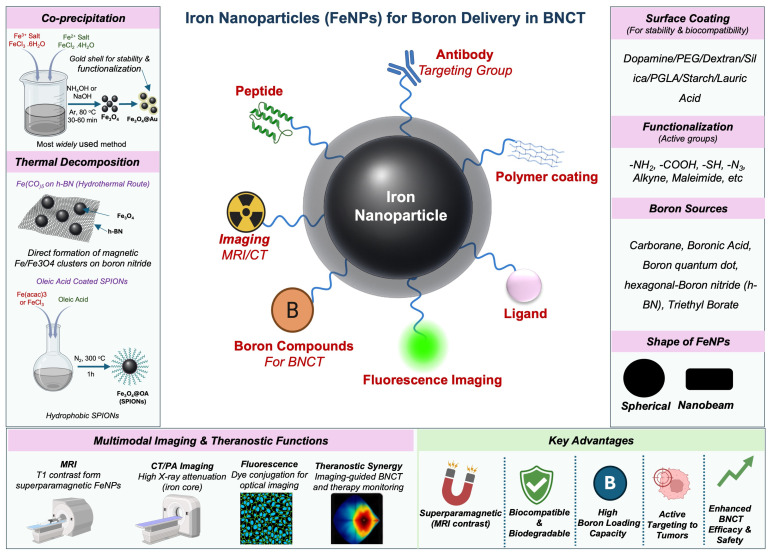
Iron oxide nanoplatforms integrating boron delivery and theranostic BNCT. (Figure was created using Biorender and Figurelab AI (pro version)).

The main attraction of iron nanoparticles is their magnetic properties. Targeted drug delivery, hyperthermia treatment, and magnetic resonance imaging (MRI) are just a few of the biomedical uses for these magnetic nanoparticles (MNPs) (Figure 4). Anticancer drugs can be directed to tumor locations in a regulated and targeted way via external magnetic fields. This magnetic guidance reduces their systemic toxicity while increasing their therapeutic effectiveness. MNPs have garnered significant interest as boron delivery vehicles in BNCT because of these benefits. Tumor-targeting ligands and boron-containing chemicals can functionalize their surfaces to mediate boron accumulation within cancer cells while also providing imaging and therapeutic capabilities. As a result, MNPs provide intriguing multifunctional platforms for enhancing BNCT treatment results and boron delivery.

In 2010, Hosmane and coworkers successfully modified MNPs by conjugating carborane cages via click chemistry. The resulting nanocomposites revealed high tumor cell accumulation under an external magnetic field, thus emphasizing their potential for BNCT, MRI, and thermotherapy applications [46]. Alexiou and coworkers reported superparamagnetic iron oxide nanoparticle (SPION)-based magnetic drug targeting (MDT) in a 2014 publication. This offers a promising strategy for boron delivery in BNCT by high boron enrichment in tumors without significant loss of function. When compared to native tissues, pilot irradiation trials revealed much higher dose deposition [47]. In 2020, Torresan et al. demonstrated the synthesis of Fe-B multifunctional nanoparticles via laser-assisted protocol. These MNPs stand as potential agents for MRI-guided BNCT that mediate customized boron imaging without the need for empirical models or radiotracers. Furthermore, they showed remarkable boron loading, good biocompatibility, and lysosomal breakdown with in vivo elimination via the liver, spleen, and renal pathways [48]. Makatsaria and colleagues published in 2024 their synthesis of magnetite-doped hexagonal boron nitride (h-BN-Fe_3_O_4_), which may represent the most suitable candidate for BNCT due to its favorable magnetic properties and stability in aqueous media [49]. 2024 also saw Wang and associates demonstrate redox-responsive boron/iron nanochains (RBNCs) that undergo tumor-specific size reduction in response to glutathione (GSH) and H_2_O_2_. This size reduction leads to controlled release of boron agents and Fe^2+^. This nanochain enhanced BNCT efficacy, while simultaneously promoting chemodynamic therapy and ferroptosis through ROS generation, GSH depletion, and GPX4 inhibition [50]. In 2025, Bekbol and coworkers developed a novel strategy using multifunctional Fe_3_O_4_ nanoparticles modified with boron and gold for cancer diagnosis and BNCT. The nanoparticles exhibited low cytotoxicity and efficient tumor cell accumulation at BNCT-relevant levels [51]. Gao and coworkers published in 2026 the synthesis of paramagnetic iron borate nanobeams (IBNBs) through a thermal decomposition protocol. Later, these were coated with silica, which improved their biocompatibility and boron uptake efficiency. These nanobeams enabled effective T1-weighted contrast by MRI. Neutron irradiation resulted in substantial tumor suppression and significantly extended the survival time [52].

Overall, iron nanoparticles are a viable platform for BNCT because of their biocompatibility, magnetic characteristics, and simplicity of surface functionalization (Table 3). They can be modified to transport boron-rich compounds and improve tumor-targeted administration by increasing permeability and their propensity for guidance in the body by external magnets. Iron-based systems also offer other benefits, such as MRI contrast capabilities, which enables theranostic applications. Given their dual function (MRI contrast agent and drug delivery platform), iron nanoparticles could be utilized for image-guided neutron irradiation and the enhancement of BNCT pharmacokinetics.

### 5.3. Boron Nanoparticles

**Figure 5:**
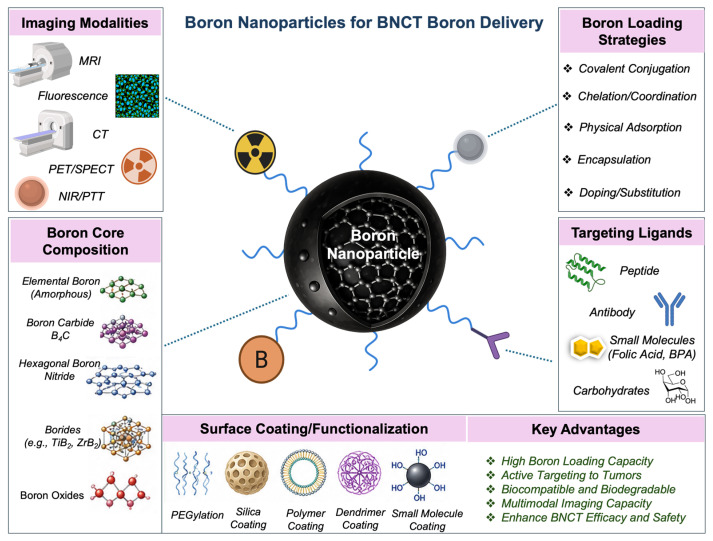
Boron nanoplatforms integrating boron delivery and theranostic BNCT. (Figure was created using Biorender and Figurelab AI (pro version)).

Boron-based nanoparticles (BNPs) are unique from the other classes in that their very structure largely consists of boron. Because they can transport large quantities of boron selectively into tumor cells, they have become attractive carriers for BNCT (Figure 5). Their size, surface characteristics, and biodistribution are precisely tunable using a variety of materials, including liposomes, polymers, AuNPs, silica nanoparticles, and MNPs. Tumor-specific absorption and intracellular boron delivery are further improved by surface functionalization using targeted ligands, antibodies, or peptides. BNPs may have several uses beyond enhancing boron accumulation, including imaging, controlled release of medication, and combinatorial therapeutic effects. Together with active targeting techniques, their increased permeability and retention (EPR) impact improves tumor selectivity and lessens toxicity to healthy organs. To address the drawbacks of traditional boron agents and enhance the therapeutic efficiency of BNCT, BNPs are being thoroughly investigated as cutting-edge boron delivery vehicles.

In 2006, Mortensen and colleagues developed amorphous boron carbide nanoparticles via the ball milling process. Irradiation studies demonstrated significant cell death, which supports the feasibility of boron carbide nanoparticles as novel agents for encapsulation in T-cells to mediate BNCT [53]. The same group also published in 2006 chemically functionalized boron carbide nanoparticles via both covalent attachment (translocation peptides and fluorescent dyes) and hydrophobic association (amphiphilic molecules). Significant intracellular boron loading was accomplished in EL4 (thymoma) and B16 (melanoma) cells. Neutron irradiation of cultures of the above cells significantly inhibited proliferation and inhibited neighboring untreated cells. Together, these findings indicate the potential of derivatized boron carbide nanoparticles for T-cell-guided BNCT [54].

After those early developments, subsequent research increasingly focused on improving boron delivery efficiency, targeting specificity and multifunctionality of boron-based nanoparticles. In more recent years, Chiang and associates documented in 2021 their synthesis of non-^10^B-enriched polymer (PEI and PEG)-coated boron carbon oxynitride (PEG/PEI@BCNO) nanoparticles for BNCT applications. PEG@BCNO nanoparticles demonstrated modest uptake (~16 µg B/g cell) and decreased cytotoxicity, whereas PEI@BCNO nanoparticles showed greater cellular uptake (~48 µg B/g cell). Neutron irradiation studies exhibited improved tumor cell killing (up to 43% cell death) compared with BPA-F, thus supporting the great potential of polymer-coated BCNO nanoparticles as affordable and efficient boron nanodrugs [55]. Kozień and colleagues reported that same year B4C-IgG nanoconjugates as targeted boron carriers capable of selective binding and internalization by phagocytic RAW 264.7 cells. This approach might improve boron delivery for BNCT, especially in tumors with low nanoparticle interactions, such as MC38 (colon adenocarcinoma) cells [56]. Kaur also reported that year the synthesis of ^10^B-enriched boron carbide nanoparticles that had a spherical shape and hexagonal structure. The nanoparticles’ low cytotoxicity, higher neutron-induced cell killing versus BPA, and blue fluorescence appropriate for cell imaging suggested strong potential for BNCT [57].

The development of targeted and multifunctional boron nanoplatforms continued in 2022. Hwang and coworkers reported a single-step microwave arcing approach for the synthesis of highly ^10^B-enriched BNPs functionalized with anti-EGFR antibodies. Designed for targeted BNCT of head and neck cancers, these nanoparticles met BNCT requirements by achieving high boron accumulation and a favorable tumor-to-blood ratio (4.27) [58]. Wang and associates then developed poly(glycerol)-functionalized ^10^B-enriched boron carbide nanoparticles as dual-function nanosensitizers for BNCT and photothermal therapy. With their significant intratumoral boron accumulation, good tolerability, and synergistic tumor elimination via BNCT + PTT, the nanoparticles showed incredible potential for cancer treatment [59].

Other studies continued to be reported throughout 2023. Kozień and associates synthesized boron carbide powders, which primarily comprise rhombohedral B_13_C_2_ and tetragonal B_48_(C_2_B_2_) phases. They demonstrated strong water dispersibility, which encouraged cellular absorption and efficient distribution in biological environments [60]. Vitali et al. utilized commercially available boron carbide nanoparticles stabilized with polyacrylic acid (PAA) and a gadolinium-rich solid phase. After fluorescent labeling, cellular imaging after BNP treatment showed rapid cellular uptake, low toxicity, and stability of their multifunctional components (boron, gadolinium, and fluorophores) during cellular internalization [61]. Zhang and colleagues reported their ^10^B-enriched hexagonal boron nitride (h-^10^BN-PG) nanoparticles grafted with poly(glycerol). These BNPs showed increased boron loading, better stealth characteristics, longer circulation, and superior tumor accumulation and penetration when compared to traditional PEGylated systems. After a single nanoparticle injection and neutron irradiation, h-^10^BN-PG stimulated antitumor immune responses and nearly eliminated tumors in preclinical mice [62].

More studies published in 2024 explored other BNP methods and platforms. Wróblewska and associates demonstrated that macrophages, especially those originating from bone marrow, are attractive carriers for boron carbide nanoparticles for BNCT. These macrophages exhibit natural tumor-homing ability and physiological relevance, as evidenced by loading with smaller B4C nanoparticles. Cell viability and motility were preserved while facilitating effective boron transport [63]. Cudziłoet and colleagues developed a unique self-sustaining combustion synthesis technique to produce nanoscale carbonated boron nitride (CBN) particles with covalently bounded amino, imino, and hydroxyl groups. The advantageous hydrophilic properties of these CBN particles enabled additional surface functionalization for the use with biological applications. These CBN nanoparticles were validated as potential agents for BNCT after tumor-targeting surface modification [64].) Demichelis and associates developed composite B_4_C-SPIONs coated with poly(acrylic acid) and functionalized with fluorophore DiI for imaging and BNCT applications. Neutron autoradiography verified the intracellular distribution of these nanostructures, which demonstrated effective absorption in HeLa cells with notable ^10^B internalization [65].

Building upon these advances, studies in 2025 emphasized receptor-targeted delivery and cell-mediated nanoparticle transport. Xu and colleagues successfully synthesized folate-functionalized boron carbide nanoparticles (with/without PEG linkers). The authors achieved up to 10% FA surface loading and long-term stability for BNPs in PBS. The nanocarriers exhibited excellent hemocompatibility, low cytotoxicity, and enhanced uptake in FR-overexpressing tumor cells [66]. Paola and associates reported tumor-infiltrating lymphocytes (TILs) as biological carriers for BNPs to improve tumor selectivity and increase T/N. Preliminary results showed that BNP-loaded TIL administration decreased tumor cell viability, and boron carbide nanoparticles were successfully loaded into lymphocytes without impacting cell survival or motility. This strategy offers a promising platform for targeted cancer treatment and nanoparticle delivery in solid tumors by combining BNCT cytotoxicity with T-cell tumor targeting [67]. Rudawska and colleagues utilized B4C BNPs targeting LDLR and EGFR receptors on cancer cells. They found that SCC-25 cells demonstrated the highest BNP internalization among those tested [68].

BNPs are densely packed with boron, thereby providing high boron concentration to targeted cells with minimal surface functionalization using targeting ligand. In comparison to traditional boron agents, their nanoscale size and surface tunability enable better tumor targeting, increased cellular uptake, and longer retention (Table 4). Furthermore, BNPs can be functionalized with targeting ligands to increase selectivity and lower systemic toxicity. On balance, BNPs may improve BNCT performance by assuring greater and more stable boron delivery into cancer cells.

### 5.4. Carbon-Based Nanoparticles

**Figure 6:**
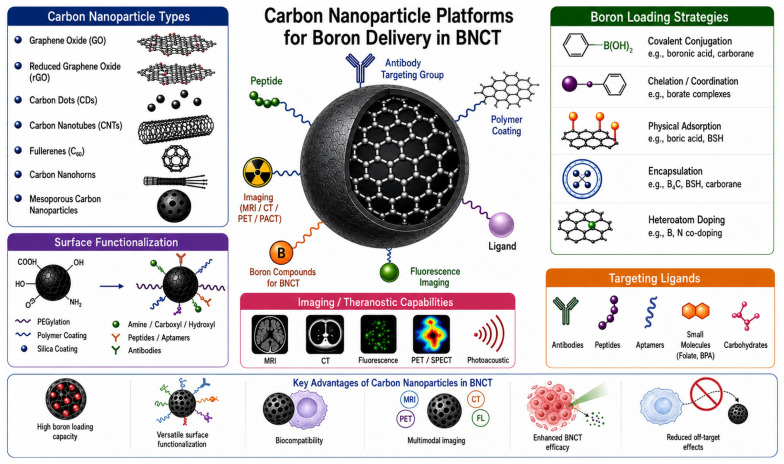
Carbon nanomaterials engineered for boron delivery in BNCT. (Figure was created using Figurelab AI (pro version)).

Carbon-based nanoparticles (CNPs) have drawn interest as prospective boron delivery vehicles for BNCT due to their high biocompatibility, chemical stability, and diverse surface chemistry (Figure 6). Among them are carbon nanotubes, graphene, fullerenes, and carbon dots. These and other CNPs are readily functionalized with boron-rich molecules and tumor-targeting ligands to mediate efficient and tumor-selective boron delivery. Their large surface area allows for significant boron loading, while their nanoscale size promotes tumor-specific accumulation via the increased permeability and retention (EPR) effect. Furthermore, CNPs can enhance imaging and photothermal characteristics, opening possibilities for theranostic applications. These characteristics make CNPs attractive platforms to increase boron delivery efficiency and thus improve the efficacy of BNCT.

The 2000s and early 2010s saw preliminary advancements in CNP chemistry, design, and conjugation techniques. In 2005, Yinghuai and colleagues demonstrated water soluble single-walled carbon nanotubes (SWCNTs) functionalized with substituted nido-carborane units as tumor-selective boron carriers for BNCT. These nanostructures’ potential for targeted boron administration was highlighted by their preferential uptake by EMT6 tumor cells [69]. In the 2010s, a report by Hwang and associates used folate-functionalized carbon nanoparticles containing ^10^B, ^157^Gd, and ^59^Co to provide a proof of concept for BNCT, Gd-NCT, and Co-NCT. Following irradiation, these tumor-targeting nanoparticles significantly reduced the clonogenicity/colony formation of cancer cells and caused BNCT-mediated cell death. Their multifunctional and magnetic characteristics also point to possible uses in concurrent cancer treatment and MRI-based diagnostics [70]. In 2013, CongXin and colleagues reported folate receptor (FR)-targeted CNPs containing ^10^B as delivery vehicles for BNCT in nonfunctional pituitary adenomas (NFPAs). Their great potential as a therapeutic approach for invasive and treatment-resistant benign tumors was demonstrated by dramatically decreased cell viability and significantly increased apoptosis [71].

From 2018 onward, this increasingly popular strategy of multifunctional and targeted nanoparticles was the focus of CNP research. Yamagami and colleagues reported in 2018 a boron-rich nanohybrid by attaching BSH-functionalized PAMAM dendrimers to single-walled carbon nanotubes (SWCNTs). The resultant SWCNT/dendrimer nanohybrid showed NIR-I to NIR-II fluorescence, which made it possible to image boron clusters in vivo and showed promise as a theranostic platform for BNCT applications [72]. In 2020, Feiner and associates created boron-rich carbon dots functionalized with tetrazine for BNCT and assessed them in a model of HER2-positive tumors. A bioorthogonal pre-targeting strategy employing trans-cyclooctene-functionalized trastuzumab greatly increased tumor accumulation despite the nanoparticles’ quick clearance and low passive tumor uptake. Their findings emphasized the potential for targeted boron delivery while minimizing off-target toxicity [73].

In 2025, publications took this trend a step further by focusing on scalability and clinical applicability. For example, Luo and colleagues developed high-boron-content carbon quantum dots (BAQDs) as a novel nanoplatform for BNCT. These 15% boron-by-weight ^10^B-enriched nanoparticles demonstrated the following: improved neutron capture efficiency, tumor-selective accumulation with perinuclear localization, and scalable production. Significant tumor cell suppression was reported after neutron irradiation of BAQD-treated triple-negative breast cancer cell lines, thus indicating potential for BNCT applications [74]. Li and associates developed ^10^B-enriched boron-nitride-doped nanographene (BNNG@PPEG) with high boron loading as a versatile platform for photothermal therapy, chemotherapy, and BNCT. The nanomaterial’s potential as an improved boron delivery system for combination cancer therapy was highlighted by its robust photothermal conversion, regulated doxorubicin release, and notable antitumor activity under both laser and neutron irradiation [75].

In summary, CNPs are emerging as adaptable carriers for BNCT because of their large surface area, high loading capacity, biocompatibility, and ability to be chemically functionalized (Table 5). Materials such as graphene, carbon nanotubes, and fullerenes may be efficiently loaded with boron-containing compounds, allowing for better tumor targeting and cellular absorption.

### 5.5. Silicon-Based Nanoparticles

**Figure 7:**
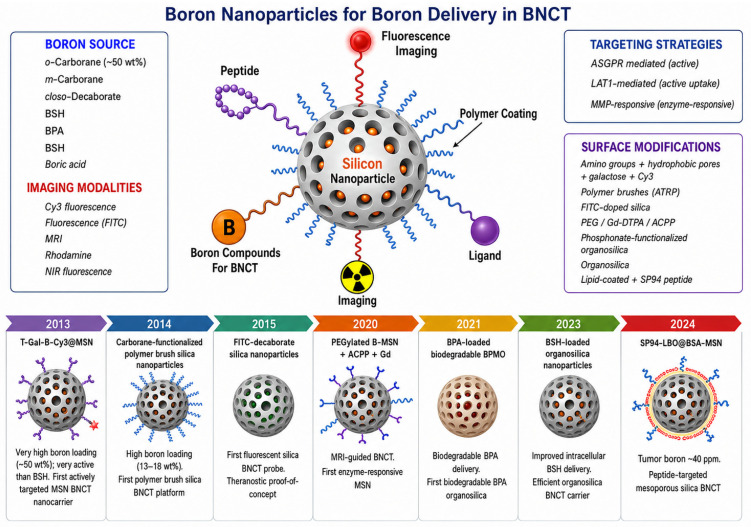
Silicon-based nanocarriers for boron delivery in BNCT. (Figure was created using Figurelab AI (pro version)).

Silicon-based nanoparticles (SNPs) are gaining more attention as potential nanocarriers for BNCT applications. Their high biocompatibility, biodegradability, and variable surface chemistry make them attractive tools for boron delivery (Figure 7). Materials like porous silicon nanoparticles and silicon quantum dots have a large surface area and porous architectures, allowing for effective loading of boron-rich compounds. Their surfaces are easily functionalized with targeting ligands, polymers, or antibodies to increase tumor-specific accumulation and intracellular delivery of boron. Furthermore, silicon nanoparticles have advantageous optical and physicochemical characteristics, making them highly useful for BNCT. Additionally, silicon nanoparticles are promising options for boosting boron delivery efficiency and BNCT therapeutic effectiveness due to their regulated breakdown for controllable release and minimal toxicity.

Research into multifunctional SNPs for BNCT was already underway in the early to mid-2010s. In 2013, Lai et al. developed multifunctional mesoporous silica nanoparticles (T-Gal-B-Cy3@MSN) loaded with o-carborane for BNCT. These luminous, galactose-targeted nanoparticles demonstrated significant boron loading (almost 60% boron-by-weight), effective cellular absorption, low cytotoxicity, and 40–50-fold greater boron delivery efficiency than BSH [76]. It was in 2014 when Brozek reported creating polymer-modified SNPs with a high boron concentration for use in BNCT. After functionalizing sub 50 nm silica particles with biocompatible polymers and carborane clusters, the SNPs demonstrated their potential as multipurpose BNCT delivery systems via greater surface targeting and imaging alterations [77]. Abi-Ghaida and colleagues reported in 2015 synthesizing luminescent surface-modified silica nanoparticle with *closo*-decaborate clusters for potential BNCT applications. These nanoparticles allowed for boron inclusion and accurate localization using fluorescence imaging while maintaining high photoluminescence. The SNPs reportedly had enhanced boron enrichment and PEG functionalization as BNCT boron carriers, despite boron loading remaining limited [78].

The 2020s kicked the earlier multifunctionality work into overdrive. Vares and colleagues demonstrated a boron delivery system for BNCT and MRI in 2020 using multifunctional fluorescent mesoporous silica nanoparticles (B-MSNs). Their B-MSNs were decorated with gadolinium and TME-activated cell-penetrating peptides. The potential of this platform for treating chondrosarcoma and other therapy-resistant malignancies was evidenced by in vitro experiments demonstrating efficient neutron-induced DNA damage and cell death in chondrosarcoma cells, including radio-resistant cancer stem cells [79]. In 2021, Tamanoi and associates developed biodegradable periodic mesoporous organosilica (^10^BPA-BPMO) nanoparticles by conjugating ^10^B-enriched BPA onto phosphonate-modified BPMO for BNCT applications. Prior to neutron irradiation, these nanoparticles showed notable tumor accumulation, effective cellular uptake, and perinuclear localization. Irradiation led to significantly inhibited tumor growth. The results show that ^10^BPA -BPMO is a potential biodegradable boron delivery system for BNCT [80]. Meanwhile, 2022 saw a report by Laird and colleagues presenting BSH-loaded periodic mesoporous organosilica nanoparticles (BSH-BPMO) via a thiol–ene grafting technique for BNCT applications. These nanoparticles demonstrated effective perinuclear localization in OVCAR8 cancer cells and tumor spheroids, improved boron uptake in comparison to BPA and BSH, and consistent boron loading without premature release. BNCT tests showed that BSH-BPMO had considerably increased treatment efficacy, underscoring their potential as sophisticated boron nanocarriers for a variety of cancer types [81]. Tang and associates put out a 2024 report on boron-containing mesoporous silica nanoparticles (SP94-LB@BA-MSN) loaded with boric acid for targeted BNCT of liver cancer. In vitro and in vivo boron uptake and accumulation were increased by lipid bilayer coating and targeting peptides, which enhanced boron retention and tumor-specific delivery. The potential of this MSN-based platform for targeted liver cancer treatment was highlighted by BNCT experiments, which showed higher therapeutic efficacy when compared to BA and BPA [82].

Overall, silicon-based nanoparticles are attractive carriers for BNCT owing to their biocompatibility, variable porosity, and large surface area for effective boron loading (Table 6). Their surfaces are readily modified with targeting ligands to increase tumor selectivity and cellular absorption, whilst their porous architectures mediate prolonged boron release and greater retention within tumor tissues.

### 5.6. Liposomes

**Figure 8:**
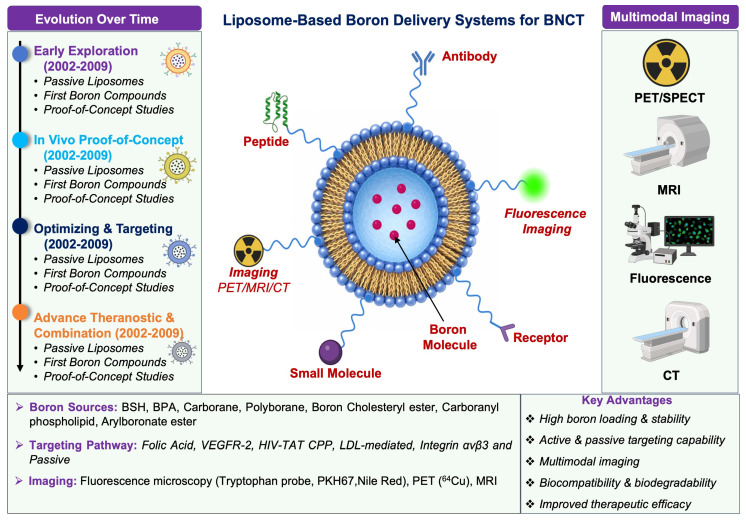
Liposome-based strategies for boron delivery in BNCT. (Figure was created using Biorender and Figurelab AI (pro version)).

These lipid-based nanoparticles are attractive in the drug delivery field for a number of reasons. In terms of BNCT, these include high biocompatibility, biodegradability, and ability to encapsulate both hydrophilic and hydrophobic chemicals. Their phospholipid bilayer structure enables easy loading of boron-containing compounds, either within the aqueous core or embedded in the lipid membrane, resulting in high boron payload delivery to tumor tissues. Liposomes can be further functionalized by adding polyethylene glycol (PEG) to increase circulation duration and targeting ligands, like antibodies, peptides, or folic acid, to produce tumor-specific accumulation (Figure 8). Their nanoscale size allows for improved permeability and retention (EPR)-mediated tumor absorption, boosting boron selectivity while lowering systemic toxicity. Due to these benefits, liposomal systems are one of the most therapeutically relevant and adaptable nanoparticle platforms for enhancing boron delivery efficiency in BNCT.

In 2002, Pan et al. showed that folate receptor (FR)-targeted liposomes could deliver extraordinarily high concentrations of boron to FR-positive tumor cells in vitro, surpassing the boron concentration needed for successful BNCT. Targeted delivery was confirmed by the fact that boron uptake was receptor-specific and could be blocked by free folate. The study emphasized the necessity for more in vivo testing for BNCT applications by highlighting boronated polyamine-loaded FR-targeted liposomes as promising boron carriers with potential for DNA-targeted delivery [83]. In 2006, Thirumamagal et al. synthesized three carboranyl cholesterol derivatives with physicochemical properties, like natural cholesterol, for BNCT applications. Without appreciably changing the stability or structure of the liposomes, one of the boronated cholesterol mimic derivatives was successfully added to non-targeted, folate receptor (FR)-targeted, and VEGFR-2-targeted liposomes. These boronated liposomes demonstrated their potential as promising boron delivery methods for tumor-targeted BNCT by exhibiting low cytotoxicity, efficient receptor targeting, and selective cellular uptake [84]. In 2009, Nakamura et al. developed a double-tailed boron cluster lipid with a B_22_H_11_S moiety as a hydrophilic functional group and its application in liposomal boron delivery systems for BNCT was assessed. Following extrusion, liposomes made with cholesterol, DSPC, PEG-DSPE, and lipid displayed a uniform size distribution, suggesting sustained vesicle production. After administering boron liposomes at a dose of 20 mg B/kg for three weeks, in vivo experiments showed minimal mortality in mice, indicating high tolerability. Additionally, following neutron irradiation in treated animals, tumor development was markedly inhibited, demonstrating the potential of these boron liposomes as efficient boron delivery vehicles for BNCT [85]. In 2009, Shirakawa et al. synthesized a boron-containing lipopeptide that was efficiently incorporated into liposomes. These boron-loaded liposomes were tested in cellular, and in vivo systems as a possible novel boron delivery system (BDS) for BNCT applications after toxicity assessment [86]. In 2010, *closo*-dodecaborate lipid-based liposomes (DSBL and DPBL) were created by Ueno et al. as innovative boron delivery methods for neutron capture therapy. This platform was special because, when exposed to neutrons, the liposomal membrane itself showed cytocidal activity. Through endocytosis, these liposomes were effectively absorbed into tumor cells without undergoing substantial disintegration. Tumor boron buildup of 22.7 ppm was shown in vivo after 20 mg B/kg of DSBL-25% PEG liposomes were administered. The promising BNCT efficacy of *closo*-dodecaborate lipid-based liposomes as dual-function boron delivery and therapeutic agents was demonstrated by the considerable tumor growth reduction that followed thermal neutron irradiation [87]. In 2012, based on advantageous boron biodistribution patterns, Herber et al. proposed that boron-bearing MAC-TAC liposomes show great promise as BNCT delivery agents. For additional testing, they suggested intravenous delivery of 18 mg B/kg followed by neutron irradiation 48–54 h after injection. The authors stressed that microlocalization and intratumoral distribution homogeneity are important factors that determine treatment efficacy beyond bulk boron concentration and cannot be properly evaluated by gross biodistribution alone. They added that research on neutron irradiation would offer both direct proof of macroscopic tissue response and crucial indirect insights into the dynamics of boron dispersion [88]. In 2013, Gifford et al. showed that therapeutically relevant levels of boron can be introduced into PC-3 prostate cancer cells for BNCT by liposome-based administration of borocaptate (BCH). After being exposed to thermal neutrons, substantial cytotoxic efficacy was demonstrated by the dramatic reduction in the clonogenic survival of targeted PC-3 cells [89]. In 2013, BSH-encapsulating 10% DSBL liposomes were created by Konganei et al. as a unique boron delivery method for neutron capture therapy. This formulation is unique because, in addition to providing encapsulated drugs, the liposomal shell itself contributes cytocidal activity. When compared to traditional BSH-loaded liposomes, the DSBL-based liposomes demonstrated significant boron loading (B/P ratio of 2.6) and effective tumor targeting, allowing for a more than fivefold reduction in the necessary liposome dose without sacrificing BNCT efficacy. BSH-encapsulating 10% DSBL liposomes were found to be an effective and promising option for BNCT applications overall. In 2013, Kueffer et al. used liposomal administration of ^10^B-enriched polyhedral borane and carborane in mouse mammary cancer models to study BNCT. After intravenous delivery, the boron-loaded liposomes produced favorable tumor-to-blood ratios and high tumor boron accumulation. When compared to untreated animals, subsequent neutron irradiation dramatically reduced tumor development, and repeated therapy further increased therapeutic efficacy. These results underlined the significance of sufficient neutron fluence for optimizing treatment outcomes and showed that liposomal boron administration in conjunction with BNCT might successfully limit tumor development [90]. In 2014, Heber et al. demonstrated that BNCT mediated by MAC-TAC liposomes produced long-lasting and efficient tumor suppression throughout a 16-week follow-up period in a hamster cheek pouch oral cancer model. Just 13% of treated cases showed tumor progression, compared to 84% and 72% of untreated and beam-only groups, respectively. The promise of MAC-TAC liposomes as efficient boron delivery agents for BNCT was highlighted by the noteworthy fact that this therapeutic efficacy was linked to minimal mucositis, decreased growth of new tumors from precancerous tissue, and the absence of considerable normal tissue harm [91]. In 2016, Takeuchi et al. created a polyborane molecule and effectively integrated it into liposomal carriers for BNCT. High tumor accumulation and selectivity were shown in biodistribution experiments, indicating its potential use for BNCT. PEGylated polyborane-loaded liposomes, with diameters of 100 and 200 nm, had better tumor-to-blood boron concentration ratios when compared to non-PEGylated formulations, indicating higher circulation and tumor targeting efficiency, according to an evaluation of the impacts of PEGylation [92]. In 2017, Kang et al. addressed the issue of inadequate tumor accumulation in BNCT and suggested a dual-targeting liposomal system modified with c(RGDyC) for selective boron delivery to glioblastoma. To increase boron uptake, the method takes advantage of the tumor cells and the highly vascularized tumor microenvironment [93]. In 2017, Takeuchi et al. created a hydrophobic polyborane molecule and effectively added it to PEGylated and naked liposomes for BNCT. Within 8–24 h after injection, these nanocarriers produced tumor boron concentrations surpassing 30 μg/g tissue, with tumor-to-blood ratios greater than five, according to in vivo biodistribution experiments. Notably, for 12 h, intratumoral boron levels in PEGylated liposomes exceeded 100 μg/g; however, subsequent leakage from tumor tissue was noted. Within four to twenty-four hours, significant boron buildup was also found in the brain. Overall, the study elucidated the biodistribution behavior of ~40 nm liposomes and supported the potential of polyborane-loaded PEGylated liposomes as effective boron delivery systems for BNCT [94]. Subsequently, Takeuchi et al. [95] created polyboranes in 2018 and effectively encapsulated them in liposomal carriers for BNCT applications. Biodistribution investigations supported the liposomes’ potential use for BNCT by confirming their high tumor accumulation and selective delivery. Specifically, after 4–24 h after treatment, liposomes made using a pH gradient loading technique demonstrated increased tumor formation. Without isotopic enrichment, these formulations were projected to generate intratumoral B concentrations of roughly 20–30 μg/g tissue, further demonstrating their potential as efficient boron delivery systems for BNCT applications [95]. In 2017, Ueda et al. reported arylboronate esters having methyl substituents at both ortho positions can be steadily integrated into lipid bilayers at high concentrations without hydrolyzing to the corresponding boronic acids. The boron loading capacity of liposomal boron delivery systems for BNCT applications can be further increased by combining this strategy with other methods that enhance the chemical stability of boron payloads within liposomal membranes [96]. In 2019, Luderer et al. developed two novel thermosensitive liposomal (TSL) formulations, B-381 and BPA-f, for boron delivery in BNCT. Both approaches showed preferential boron buildup in tumors in vivo and hyperthermia-triggered release. Notably, when compared to BPA-f formulations, B-381 TSLs demonstrated extended tumor retention up to 24 h. This work establishes a basis for future clinical development of thermosensitive boron-loaded liposomes for BNCT applications and is the first demonstration of boron delivery using TSLs [97]. In 2019, Olusanya et al. reported *o*-carborane-loaded DPPC and DSPC liposomes synthesized in thin films produced stable ~80–100 nm tiny unilamellar vesicles with good monodispersity (PDI < 0.5), with minor size increases upon increased boron loading. Despite the modest colloidal stability indicated by ζ-potential values, AFM verified that the formulations improved after storage and re-probing and were structurally intact for long periods of time. Using ICP-MS analysis and calcein retention, high serum stability and quantitative boron incorporation were confirmed. All things considered, these liposomes demonstrated robust structural integrity and consistent boron loading, indicating their potential as BNCT delivery platforms [98]. In 2019, Takeuchi et al. prepared polyborane-encapsulated PEGylated liposomes employing a post-insertion methodology, achieved comparable therapeutic effectiveness with half the quantity of PEG lipid compared to traditional pre-PEG procedures. Pre-PEG and post-PEG liposomes did not differ significantly in surface PEG density or FALT analysis. Studies on cellular absorption, in vivo biodistribution, and in vitro cell survival further verified that both formulations performed similarly. Overall, the study backs up the post-insertion method as a successful PEGylated boron liposome design technique for BNCT applications [99].

In 2020, Lee et al. successfully loaded water-soluble *nido*-carborane anions at high concentrations into PEGylated liposomes. With effective cytoplasmic localization in tumor cells, these boronated liposomes demonstrated deep and consistent tumor penetration. The powerful therapeutic potential of PEGylated boron-loaded liposomes was demonstrated in BNCT tests where a single administration coupled with 20 min of neutron irradiation produced nearly total suppression of tumor growth [100]. In 2021, Kanygin et al. demonstrated that fluorescence microscopy was a quick and efficient method for monitoring the intracellular location of fluorescently labeled liposomes. In U87 glioma cells, liposome-encapsulated lipophilic and water-soluble indicators were distributed uniformly. When compared to normal cells, PEGylated liposomes showed noticeably greater tumor accumulation; SW-620 and U87 cell lines showed particularly strong absorption. Peak tumor accumulation in an orthotopic U87 brain tumor model was many times more than in normal brain tissue and happened about six hours after injection. Overall, the work emphasized fluorescence microscopy as a helpful screening method for assessing tumor targeting and liposomal delivery in the creation of boron compounds [101]. In 2021, Shirakawa et al. created a novel boron lipid, PBL, as well as PBL-based liposomes in which boron was positioned in the outer aqueous phase without interfering with medications contained in the inner core for BNCT. Like PEGylated liposomes, these liposomes were anticipated to show increased tumor formation and longer blood circulation while enabling the co-encapsulation of anticancer drugs for combination therapy. PBL liposomes were suggested as prospective boron delivery agents for BNCT applications due to their high boron payload and multifunctional architecture [102]. In 2021, Zhang et al. used the high neutron capture cross-section of ^10^B to create a thermal neutron-sensitive composite liposomal system. To facilitate magnetic separation and analyte enrichment after neutron irradiation, the liposomes co-encapsulated Fe_3_O_4_@OA nanoparticles, methylene blue (MB), and anti-BSA antibodies. Thermal neutron treatment resulted in the controlled release of MB and antibodies. Spectrophotometry and SPR quantitative analysis of the released components revealed a dose-dependent response. All things considered, this technology shows a sensitive and adaptable platform for thermal neutron detection. In 2022, Li et al. reported the development of boronsome, a boron-enriched lipid bilayer nanoplatform for combinational BNCT applications. Carborane-functionalized phospholipids produced persistent biomimetic nanovesicles that could effectively and selectively transfer boron to tumor cells without causing considerable toxicity, according to molecular dynamics simulations and experimental characterization. Furthermore, by combining BNCT-induced DNA damage with the inhibition of DNA repair mechanisms, boronsome-mediated co-delivery of PARP inhibitors showed synergistic anticancer efficacy. This novel chemoradiotherapeutic method offers a viable way to improve BNCT effectiveness and make future clinical translation easier [103].

Liposomes are well-known nanocarriers for BNCT because of their high biocompatibility, low toxicity, and ability to encapsulate high concentration of boron compounds inside their lipid bilayer or aqueous core. Their surface can be easily functionalized with targeting ligands like antibodies or peptides to improve tumor-specific delivery and cellular absorption. Liposomes also increase boron uptake and retention in tumor tissues, lowering systemic exposure and off-target effects. Overall, liposomal systems offer a therapeutically relevant and adaptable platform for increasing boron delivery efficiency in BNCT, with significant potential for further use in cancer treatment (Table 7).

### 5.7. Polymer-Based Nanoparticles and Micelles

**Figure 9:**
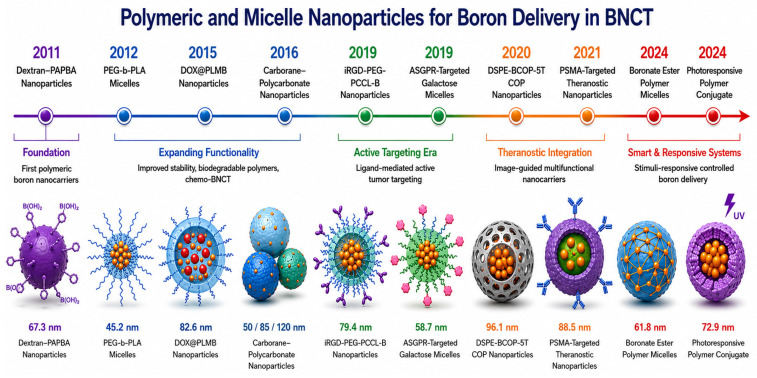
Development of polymeric and micelle-based boron delivery for BNCT. (Figure was created using Figurelab AI (pro version)).

Polymer nanoparticles and polymeric micelles are primarily made of amphiphilic block copolymers, which self-assemble into core–shell structures. The hydrophobic core may enclose boron-rich chemicals, while the hydrophilic shell improves water stability and systemic circulation. Their surfaces may be readily modified with polyethylene glycol (PEG) and tumor-targeted ligands, allowing for better pharmacokinetics and selective tumor accumulation through active targeting and the increased permeability and retention (EPR) effect (Figure 9). In addition, polymer-based nanocarriers have regulated release behavior, which can improve intracellular boron availability at the tumor location. They have received much interest as effective boron delivery vehicles for BNCT because of their structural diversity, biocompatibility, and capacity to transport large drug loads.

In 2011, Zhang et al. developed dextran-PAPBA nanoparticles through aqueous polymerization of APBA in the presence of dextran, producing stable and small-sized nanoparticles with improved structural integrity. Doxorubicin (DOX) was effectively encapsulated by these nanoparticles, which also demonstrated prolonged, pH-responsive drug release while preserving DOX pharmacological activity. Dextran-PAPBA nanoparticles show excellent promise for combination chemotherapy and BNCT applications in cancer treatment due to their variable boron content, improved membrane-crossing capabilities, and regulated drug administration [104]. In 2012, Sumitani et al. developed polymeric micelles (PMs) comprising acetal-PEG-b-PLA-MA and VB-carborane to prevent nonspecific boron release during circulation. In comparison to non-polymerized micelles, PMs effectively loaded boron compounds and, by covalent bonding, totally inhibited boron leakage in serum, leading to increased tumor formation and longer blood circulation. Crucially, the ^10^B-enriched PMs showed exceptional therapeutic effectiveness under BNCT and were completely removed in 7 days, underscoring their potential as straightforward, therapeutically adaptable, and selective boron carriers for cancer therapy [105]. In 2015, Xiong et al. created DOX-loaded carborane-conjugated polymeric nanoparticles (DOX@PLMB) to combine chemotherapy and BNCT. High boron stability, low leakage, extended blood circulation, and increased tumor growth were all demonstrated by the self-assembled nanoparticles. Through carborane–DOX interactions, DOX was well encapsulated and exhibited pH-responsive release. While combination DOX@PLMB treatment with thermal neutron irradiation produced improved therapeutic efficacy over chemotherapy or BNCT alone, in vivo investigations showed greater and sustained tumor drug accumulation compared to free DOX. These results demonstrate the potential of PLMB nanoparticles as a low-toxicity platform for effective boron administration and combination chemoradiotherapy [106]. In 2016, Xiong et al. used ring-opening polymerization to create amphiphilic biodegradable carborane-conjugated polycarbonate nanoparticles for BNCT. Nanoparticles of different sizes were produced by adjusting the composition of the polymer; smaller particles (PN50) demonstrated better cellular uptake, tumor formation, and decreased protein adsorption and liver clearance in comparison to their bigger counterparts. Size-optimized biodegradable polycarbonate nanoparticles are promising boron carriers for BNCT, as demonstrated by the maximum therapeutic efficacy and lowest systemic toxicity when PN50 was paired with thermal neutron irradiation [107]. To produce boron-rich self-assembled nanocarriers for BNCT, Yoneoka et al. created poly(NIPAAm-block-NIPAAm-co-PBA), a thermo-responsive boron-containing diblock copolymer, using RAFT polymerization in 2018. The copolymer formed nanomicelles and ~80 nm core–shell nanoparticles with substantial boron loading because of temperature-dependent phase changes. These water-dispersible, size-controlled nanoparticles have shown promise as innovative boron nanocarriers for BNCT by efficiently concentrating boron within their cores and demonstrating potential for tumor targeting via the enhanced permeability and retention (EPR) effect [108]. In 2019, Chen et al. developed an iRGD-functionalized PEG-PCCL polymeric nanoparticle system for combining BNCT and chemotherapy by co-delivering (boron sources) and DOC. The ~25 nm biodegradable nanoparticles demonstrated improved cellular absorption (209.83 ng B/10^6^ cells) in integrin/NRP-1 overexpressing cells, as well as effective nuclear delivery of DOX and cytosolic boron localization. Strong potential for synergistic BNCT–chemotherapy was highlighted by the system’s increased tumor accumulation and extended circulation in A549 tumor models. Furthermore, it has been demonstrated that tumor vascular normalization (e.g., using Endostar) enhances the delivery of nanoparticles, thus supporting its function in improving therapeutic efficacy in resistant cancers [109]. In 2019, Zhang et al. developed a PEGylated galactose micelle containing carborane clusters for targeted BNCT against hepatocellular cancer (HCC) via ASGP-R receptor recognition. When compared to BSH, the micelles showed improved HepG2 cell uptake and selectivity, as well as reduced cytotoxicity and DNA double-strand breaks that induced cancer cell demise. The approach demonstrated low systemic toxicity, good tumor targeting, and effective body clearance in vivo. This PEGylated galactose micelle is a potential boron delivery platform for targeted BNCT and receptor-mediated medication administration in HCC therapy, even though receptor heterogeneity and irradiation issues still exist [110].

In 2020, Shi et al. synthesized a nanoscale covalent organic polymer (COP) functionalized for PET/CT image-guided BNCT. The carborane-loaded BCOP-5T nanoparticles coated with DSPE-PEG demonstrated enhanced stability, biocompatibility, and effective boron transport. The technique allowed for theranostic monitoring and in vivo PET imaging after radiolabeling with copper-64. DSPE-BCOP-5T’s promise as an image-guided BNCT nanoplatform for cancer therapy was demonstrated by the considerable suppression of tumor growth in 4T1 tumor-bearing mice when paired with thermal neutron irradiation [111]. In 2021, Vedelago et al. developed a unique boron-enriched three-dimensional polymer network crosslinked with boric acid molecules as a possible material for boron-based radiation therapies, such as proton–boron fusion therapy and BNCT. The study used Monte Carlo simulations with MCNP and FLUKA programs and mechanical property analysis to assess the material’s reaction to thermal neutron irradiation. The material’s intriguing interaction with mixed radiation fields and its potential for future cancer therapy applications were supported by the high agreement between experimental and modeling results [112]. In 2021, Kim et al. exploited PBA-installed amphiphilic block copolymer nanoparticles for BNCT to show the possibility of sialic acid-targeted boron administration. When compared to BPA, the PBA-decorated nanoparticles (NanoPBA-I and NanoPBA-II) demonstrated quick and stable binding to the membrane of cancer cells and markedly increased cellular absorption. Because of its efficient tumor targeting and advantageous intracellular localization, NanoPBA-I demonstrated anticancer activity in a B16 melanoma model that was on par with BPA despite being given at a 100-fold lower dose. These results demonstrate the potential of active-targeting polymeric nanoparticles as next-generation BNCT agents [113]. In 2021, Meher et al. developed PLGA-b-PEG theranostic nanoparticles functionalized with DFB chelators and PSMA-targeting ACUPA ligands for concurrent PET imaging and boron delivery in prostate cancer BNCT. Due to quick carborane release and inadequate serum stability, ACUPA-conjugated nanoparticles demonstrated PSMA binding in vitro and obtained a twofold greater uptake in PSMA-positive tumors, but overall tumor accumulation and boron delivery remained restricted. Despite these drawbacks, the work offers insightful information for future PSMA-targeted boron delivery system design and improvement for BNCT [114]. In 2022, Chan et al. showed that PVA/BA-crosslinked nanoparticles function as effective boron carriers for BNCT, outperforming boronophenylalanine in vivo with a 44.29% reduction in tumor size and reaching ~70-fold higher tumor boron uptake than the therapeutic threshold. All things considered, these nanoparticles exhibit great promise for successful BNCT-based oral cancer treatment [115]. In 2024, Fu et al. created a boron-rich *m*PEG-b-PBE36 block copolymer using ATRP that self-assembles into stable micelles that are around 43 nm in size for nanotherapeutic applications. The micelles exhibit better therapeutic efficacy with higher tumor cell death and tumor growth delay following neutron irradiation, as well as high biocompatibility and significantly increased boron uptake (38-fold higher than BPA). All things considered, they are highly promising, and safe nanocarriers for effective boron delivery in BNCT [116].

Polymeric nanoparticles and micelles are highly attractive nanocarriers for BNCT because of their great biocompatibility, structural plasticity, and ability to effectively load boron-containing compounds. The enhanced permeability and retention (EPR) effect allows these systems to be tailored for regulated drug release, better circulation stability, and increased tumor accumulation. Surface functionalization with targeting ligands enhances cellular uptake and selectivity for cancer cells. Overall, polymer-based nanoparticles and micelles improve boron transport efficiency and therapeutic effectiveness in BNCT (Table 8).

### 5.8. Nanoconjugates

**Figure 10:**
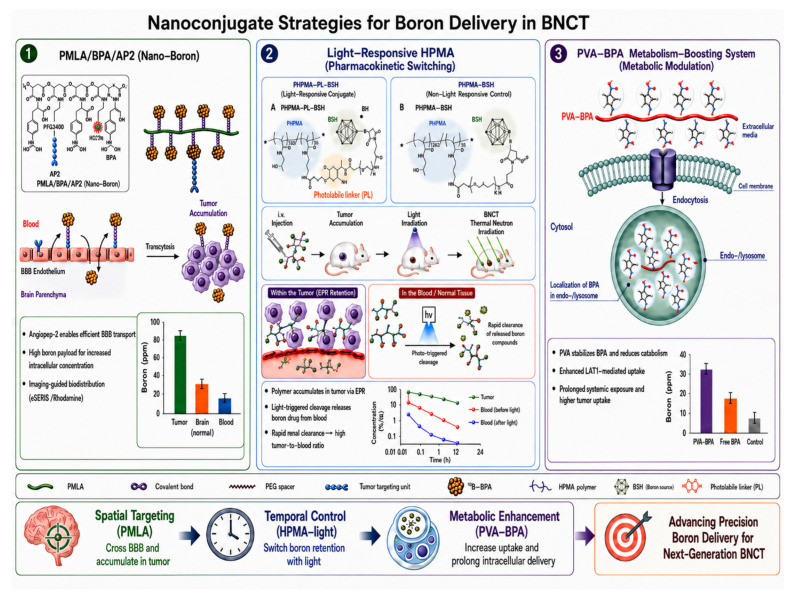
Nanoconjugate boron carrier for targeted BNCT applications. (**A**) Schematic layout of the components of this light-responsive PHPMA-PL-BSH conjugate. Note photolabile section highlighted with manila accent glow. (**B**) Schematic layout of the components of this non-light-responsive PHPMA-BSH conjugate, which acts as a control versus the compound in (**A**). The *’s in both subpanels indicate additional monomers in the poly-HPMA backbone at either end of the monomer shown. (Figure was created using Figurelab AI (pro version)).

Nanoconjugate are nanosized drug delivery platforms that consist of drug molecules or covalently conjugated drug moieties. In recent years, there has been growing interest in these nanomaterials/nanomedicines for BNCT and other therapies due to their enhanced solubility, stability, and tunable release profile for improved efficacy and safety (Figure 10). Many nanoparticles and formulations in the preclinical stage suffer from burst release (rapid release all at once or over a short clinically irrelevant time period) of drug molecules in physiological condition, which is a major problem in designing drug delivery strategies. Nanoconjugates offer covalent attachment of drug molecules, which makes them less susceptible to burst release, thus providing an attractive alternative for boron delivery.

In 2020, Nomoto et al. revealed that poly(vinyl alcohol) (PVA) may form reversible boronate ester complexes with BPA in aqueous environments, resulting in a PVA-BPA complex. This method was proven to improve tumor targeting by allowing LAT1-mediated cellular absorption of BPA while decreasing its efflux from cancer cells. In vivo tests demonstrated that PVA-BPA outperformed the currently utilized fructose-BPA formulation in terms of tumor formation and retention, as well as with quicker clearance from the circulation and normal tissues. Collectively, these qualities resulted in greatly improved treatment effectiveness in boron neutron capture therapy (BNCT) [117].

To overcome the primary drawback of BNCT, ineffective and non-selective boron delivery, Patil et al. suggested a nano-boron platform in 2024, especially for the clinically intractable GBM. To allow for blood–brain barrier penetration, the system comprises a PMLA-based nanocarrier coupled with isotopically enriched BPA and an angiopep-2 (AP2) peptide for targeting the GBM-overexpressed low-density lipoprotein receptor protein 1 (LRP1). This formulation increases therapeutic potential while preserving low toxicity and high solubility by enabling targeted and localized intracellular delivery of ^10^B to tumor cells at lower concentrations. Overall, by enhancing selective boron accumulation in malignant tissues, nano-boron offers a viable approach to improve BNCT effectiveness for GBM and other cancers [17]. In 2024, Tokura et al. developed a light-controlled polymer–drug conjugate (PHPMA-PL-BSH) for BNCT that uses photolabile linker to regulate boron pharmacokinetics and improve tumor selectivity. The system initially exhibits a low tumor-to-blood (T/B) ratio but passively accumulates in tumors following intravenous delivery. Tumor-retained conjugates remain after targeted light irradiation, while boron is selectively removed from circulation and quickly eliminated by renal excretion, greatly raising the T/B ratio above the clinical threshold. This temporally controlled method shows a unique light-responsive methodology for BNCT drug delivery systems by increasing intratumoral boron concentration and improving anticancer activity under neutron irradiation [118].

Nanoconjugates are emerging as effective boron delivery vehicles for BNCT because of their great structural tunability and strong ability to covalently bind boron-rich molecules (Table 9). These conjugates provide precise control over boron loading, increased water stability, and active tumor targeting as well as passive (EPR effect). Their favorable pharmacokinetic properties coupled with rapid clearance from the blood allow them to generate greater tumor-to-healthy tissue contrast for enhanced tumor selectivity and safety.

### 5.9. Dendrimers

**Figure 11:**
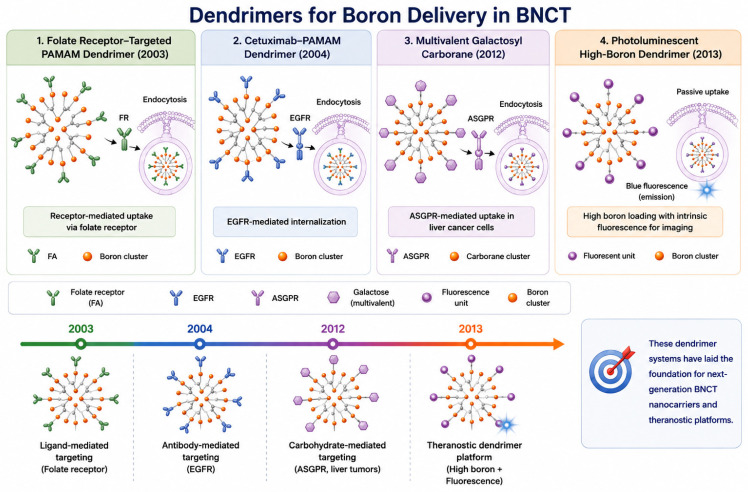
Dendrimer platforms for targeted boron delivery in BNCT. (Figure was created using Figurelab AI (pro version)).

Dendrimers are highly branched, monodisperse macromolecules that have gained researchers’ attention as enhanced boron delivery vehicles for BNCT due to their well-defined architecture and high density of functional surface groups. Their unique tree-like structure allows for the conjugation of numerous boron-containing clusters at the terminal functional sites, resulting in very high boron loading per molecule. Furthermore, dendrimers could be designed across multiple generations to optimize size, solubility, and biodistribution, and their surfaces can be changed with polyethylene glycol (PEG), antibodies, peptides, or other targeting ligands to improve tumor selectivity. Their nanoscale dimensions also allow for increased EPR-mediated tumor accumulation. Dendrimers, with their high boron payload capacity, structural tunability, and variable surface chemistry, are one of the most promising nanoplatforms for increasing boron delivery efficiency and therapeutic efficacy in BNCT (Figure 11).

As targeted boron delivery methods for BNCT, Shukla et al. created chromophorically labeled PEGylated and folic acid (FA)-conjugated boronated dendritic entities (BDEs) in 2003. Reduced hepatic absorption of PEGylated G3-BDEs suggested their better biodistribution and decreased affinity for the reticuloendothelial system (RES). Compared to previous BDE–antibody and BDE-EGF conjugates, FA-conjugated/PEGylated BDEs demonstrated receptor-mediated absorption in folate receptor-positive KB cells and improved tumor selectivity. High liver and kidney accumulation, however, continued to be a drawback, underscoring the necessity of more PEGylated receptor-targeted BDE modification for enhanced BNCT efficacy [119]. In 2004, Wu et al. developed an EGFR-targeted boron delivery system by conjugating the anti-EGFR monoclonal antibody cetuximab (C225) with a highly boronated fifth-generation polyamidoamine (PAMAM) dendrimer (G5-B1100). The produced compound, C225-G5-B1100, retained a high receptor-binding affinity despite including over 1100 boron atoms per cetuximab molecule. The compound significantly increased boron accumulation in EGFR-expressing tumors after intratumoral treatment in rat glioma models as compared to wild-type tumors and unconjugated dendrimer controls, demonstrating effective receptor-mediated targeting. These findings showed that cetuximab-mediated boron administration is feasible and emphasized the potential of EGFR-targeted nanoconjugates to improve the therapeutic effectiveness and selectivity of BNCT in brain cancers that express EGFR [120]. In 2012, Lai et al. synthesized a multivalent galactosyl carborane derivative, known as dendritic glyco-borane (DGB 10), as a targeted boron delivery agent for BNCT. In HepG2 liver cancer cells, the chemical exhibited increased boron absorption and, when exposed to neutron radiation, showed approximately ten times more cell-killing effectiveness than BSH. These results demonstrate DGB 10 has potential as a cell-targeting boron carrier to increase the efficacy of BNCT [121]. In 2013, Gonzalez-Campo et al. developed a new class of star-shaped and dendritic carborane-containing molecules based on a fluorescent 1,3,5-triphenylbenzene core with three or nine terminal carborane clusters. The thermal stability of these boron-rich compounds was good; three-cage star-shaped derivatives were more stable than nine-cage dendrimers. Each molecule showed comparatively large quantum yields and blue-violet photoluminescence without total fluorescence quenching. Electrochemical investigations revealed that the fluorescent core and carborane clusters had little electrical contact, maintaining their optical characteristics. Their high boron concentration and fluorescence properties point to a promising future for biological applications and BNCT [122].

Dendrimers are very promising nanocarriers for BNCT because of their well-defined branching architecture and abundance of surface functional groups, which enable high-density boron loading. Their nanoscale size and precise molecular structure allow for rapid cellular absorption and improved tumor targeting, particularly when paired with targeting ligands. Dendrimers significantly increase the solubility, stability, and controlled administration of boron agents, resulting in increased tumor boron buildup and less off-target effects (Table 10).

### 5.10. Critical Comparison of Nanocarrier Platforms

A comparison of nanocarriers for BNCT demonstrates that each platform has certain benefits but also some key translational limits. Inorganic systems, such as gold, iron oxide, boron, carbon, and silica nanoparticles, are often characterized by strong structural stability, multifunctionality and possibilities for imaging-guided BNCT. Gold nanoparticles are especially interesting due to their biocompatibility, simple surface modification and theranostic potential; however, their boron loading is mostly restricted to surface conjugation and long-term retention is a concern. Iron oxide nanoparticles may be seen via MRI and guided magnetically, although clinical translation may be limited by aggregation, liver/spleen buildup, and complicated production.

The maximum inherent boron density, and hence the lowest need of significant chemical conjugation, may be obtained by using boron-rich nanoparticles, such as B4C, BCNO, and h-BN. However, poor aqueous dispersibility, aggregation, and repeatable functionalization are still key obstacles. Carbon-based systems possess substantial surface area, photothermal characteristics and fluorescence imaging capacity; however, problems related to clearance, cytotoxicity, and tumor formation are varied and need careful consideration. Silica-based NPs have variable porosity and high loading of boron, although traditional mesoporous silica may not be biodegradable and well-validated in vivo.

Organic and soft nanocarriers, such as liposomes, polymeric nanoparticles, micelles, nanoconjugates, and dendrimers, often have higher biocompatibility, flexible surface engineering, and increased control over drug release. Liposomes are one of the most therapeutically recognized platforms that can encapsulate both hydrophilic and hydrophobic boron agents; however, the constraint of boron leakage and formulation complexity still exists. Polymeric nanoparticles and micelles may provide pH-responsive release, ligand modification and combination treatment, although burst release and serum instability may limit efficacy. Nanoconjugates allow for controlled covalent boron conjugation and less premature release, although they need more sophisticated manufacturing and have less verified in vivo investigations. Dendrimers provide great boron loading and defined molecular structure, but their multi-step manufacturing and liver/kidney accumulation hinder their translational potential.

In general, none of the nanocarrier platforms can meet all of BNCT’s standards. Boron-rich inorganic nanoparticles give higher boron density. Liposomes, polymers, and nanoconjugates enable improved biocompatibility and targeted distribution. Gold, iron oxide, silica, and carbon platforms are particularly suited for theranostic applications, as they can couple boron administration with imaging or radiosensitizing activities. Hence, the future of BNCT nanocarrier design will likely rely on hybrid systems that can incorporate high boron payloads, active tumor targeting, imaging capabilities, controlled release, biodegradability, and low systemic toxicity into a single optimized platform. Table 11 presents a comparative summary of the nanocarriers discussed so far, as well as their therapeutic performance for BNCT.

## 6. Conclusions

This review highlights the significant progress made in the development of nanocarrier strategies for BNCT. Advances in gold, iron, boron, carbon, and silicon nanoparticles; liposomes; polymeric nanoparticles; micelles; nanoconjugates; and dendrimer nanosystems have demonstrated the potential to overcome key limitations associated with conventional boron delivery agents, particularly in terms of tumor targeting, boron loading, and pharmacokinetics. Among these, multifunctional and hybrid nanocarriers integrating targeting ligands and controlled release mechanisms have shown the most promise in improving therapeutic outcomes. Despite these advancements, several challenges remain, including issues related to toxicity, scalability, and regulatory approval. Addressing these barriers will be essential for translating nanocarrier-enabled BNCT from preclinical research to routine clinical application. Looking forward, the integration of personalized medicine approaches, combination therapies, and next-generation smart nanocarriers is expected to further enhance the precision and effectiveness of BNCT. Collectively, these developments position nanocarrier-based BNCT as a promising frontier in targeted cancer therapy, with the potential to significantly improve patient outcomes in the era of precision oncology.

The analysis of the examined works reveals that efficient BNCT nanocarriers need the harmonious incorporation of several design elements instead of the enhancement of a solitary parameter. Elevated boron loading is inadequate without effective intracellular transport, and robust tumor targeting cannot guarantee treatment success if boron retention is suboptimal. Consequently, forthcoming nanocarriers must integrate substantial boron payloads, tumor selectivity, intracellular localization, biodegradability, and minimal systemic toxicity. An optimal BNCT nanocarrier should possess a particle size of around 50–150 nm, a near-neutral or slightly negative surface charge, significant physiological stability, and active targeting functionality via receptor-specific ligands. Multifunctional designs that include imaging, controlled release, or combination treatment might enhance translational potential.

The creation of a protein corona is a significant difficulty, as adsorbed serum proteins may obscure targeting ligands, modify surface characteristics, enhance clearance, and diminish active targeting in vivo. To mitigate these effects, contemporary boron nanocarriers are designed with stealth coatings such PEG, zwitterionic polymers, poly-saccharides, or biomimetic membranes. Regulated ligand density, appropriate spacer length, and robust conjugation chemistry contribute to maintaining ligand accessibility, enhancing circulation, facilitating blood–brain barrier or tumor barrier transit, and increasing boron accumulation. The area is advancing towards hybrid, multifunctional nanocarriers that integrate complementary material benefits and may more effectively satisfy the biological and clinical demands for BNCT translation.

## Figures and Tables

**Table 1:** Comparison between recent BNCT nanocarrier reviews and the present review.

Review	Publication Year	Main Focus	Comparative Analysis	Translational Perspective	Critical Evaluation	Present Review Advantages
IJN (10.2147/IJN.S581215) [7]	2025	General overview of boron nanocarriers	Limited	Moderate	Moderate	Our review provides systematic comparison of nanocarrier performance and physicochemical determinants.
Theranostics (10.7150/thno.126209) [8]	2025	Nanotechnology and BNCT advances	Limited	Moderate	Moderate	We critically compare targeting efficiency, BBB penetration, boron accumulation, and therapeutic outcomes.
Exploration (10.1186/s12645-025-00324-3) [9]	2025	Emerging boron delivery systems	Limited	Limited	Moderate	We integrate translational barriers, regulatory considerations, theranostics, and future design principles.
Present Review	2026	Focused review of over 91 high-impact studies	Comprehensive	Comprehensive	Extensive	Critical comparison of nanoplatforms, physicochemical determinants, biological barriers, therapeutic efficacy, translational readiness, and future perspectives.

**Table 2:** Gold nanoparticle-based boron delivery platforms for BNCT, including surface engineering, multifunctionality, and biological applications.

Size (nm)	Zeta (mV)	Boron Source	Surface Chemistry	Targeting Strategy	Imaging	In Vitro	In Vivo	Advantages	Limitations	Ref.
140 ± 5	+25 ± 3	BPA	PAH/PSS multilayers, FITC, folic acid	Folate receptor	Fluorescence	JHH6, HL60, MDA-MB-231	-	First multifunctional targeted AuNPs for BNCT	No in vivo validation	[34]
10–19	-	*ortho*-Carborane	PEG350/550/2000, dendritic thiols, Au–S click chemistry	-	-	-	-	Extremely high boron loading	No biological evaluation	[35]
9.4	≈−25	*nido*-Carborane	Glutathione-coated Au nanoclusters	Passive (EPR)	Fluorescence	HeLa, U87, L02	HeLa xenograft	Imaging-guided boron delivery	No therapeutic efficacy evaluated in study	[36]
30–35	−22 ± 3	COSAN	PEG5000 + COSAN-SH	Passive (EPR)	PET/CT (^124^I)	-	HT1080 xenograft	PET-guided biodistribution	Low tumor accumulation	[37]
54–58	−9 ± 2	Carborane derivative	PEG6000 + anti-HER2 antibody	HER2 receptor	SPECT/CT (^123^I)	N87	N87 & SASVO3 xenografts	High tumor boron accumulation (>200 μg/g)	No neutron irradiation	[38]
27–40	-	COSAN	PEG + tetrazine click chemistry	HER2 bioorthogonal pre-targeting	PET/CT (^64^Cu)	BT-474	BT-474 xenograft	Novel pre-targeting strategy	Limited tumor retention	[39]
~40	−23 ± 3	COSAN	PEG + COSAN-SH	Passive (EPR)	PET/CT (^64^Cu)	MKN45, HDFa	MKN45 xenograft	Combined BNCT and photothermal therapy	Low tumor boron concentration	[40]
-	-	BPA	Glycylglycine-coated AuNPs	Passive of AuNPs and LAT1 of BPA.	Gamma spectroscopy	T98G	-	Real-time BNCT dosimetry	Not a boron carrier	[41]
160 ± 51	-	BC-EG-SH	PLGA encapsulation + Gd + AuNP	Passive (EPR)	MRI	N87	N87 xenograft	MRI-guided boron delivery	No neutron therapy	[42]
~30	-	-	PLA + Au–cysteine complex	-	-	U251, SW620	-	Implantable biodegradable dosimetry scaffold	Not a boron carrier	[43]
12 nm (PEG); 150 nm (Dextran)	≈−18 (PEG-coated)	Boron-doped Au alloy	PEG or Dextran coating	-	CT	B16, A375, PC3	-	Combined BNCT, XRT, and CT imaging	No in vivo validation	[44]
45.6	−8.45	BSH	PEG + cRGD peptide	Integrin αvβ3	CT	GL261	GL261 mice	Highly efficient glioblastoma targeting	No neutron therapy	[45]

**Table 3:** Iron oxide nanoparticle systems investigated for BNCT, focusing on boron delivery, imaging capability, and therapeutic outcomes.

Size (nm)	Zeta (mV)	Boron Source	Surface Chemistry	Targeting Strategy	Imaging	In Vitro	In Vivo	Advantages	Limitations	Ref.
~100	-	Carborane	Starch–carborane	Magnetic	MRI potential	-	BALB/c	First magnetic boron nanocarrier	No therapeutic evaluation	[46]
80–100	-	Boronic acid	Lauric acid	Magnetic	-	-	Phantom	Demonstrated BNCT dosimetry compatibility	No biological validation	[47]
64 ± 40	-	Fe–B alloy	PVP	Passive	MRI	L929, B16, 4T1	Healthy mice	MRI-visible multifunctional boron carrier	No BNCT	[48]
120–170	≈+30	Carborane	Gd-DTPA–carborane	Magnetic	MRI	T98G	-	Dual boron–gadolinium theranostic platform	No in vivo BNCT	[49]
127	+34.57	Boron quantum dots (BQDs)	Fe^3+^-assembled BQD	Redox-responsive, EPR-mediated targeting	NIR fluorescence imaging	4T_1_ breast cancer	BALB/c 4T_1_ tumor-bearing mice	First redox-responsive BNCT/CDT/ferroptosis nanoplatform	No active targeting; limited preclinical validation	[50]
689 ± 123 nm	−20.0	Carborane	Fe_3_O_4_@Au–carborane	Passive	MRI + Au	U251MG	-	Fe_3_O_4_@Au multifunctional BNCT platform	No in vivo therapy	[51]
58.8	-	Fe_2_B_2_O_5_	Fe_2_B_2_O_5_@SiO_2_	Intratumoral	T_1_ MRI	B16F10	C57BL/6	MRI-guided BNCT with marked tumor suppression	Intratumoral injection	[52]

**Table 4:** Boron-based nanocarriers for BNCT, emphasizing material design, targeting approaches, and preclinical findings.

Size (nm)	Zeta (mV)	Boron Source	Surface Chemistry	Targeting Strategy	Imaging	In Vitro	In Vivo	Advantages	Limitations	Ref.
~73 (PCS)	NR	Natural/^10^B-B_4_C	Bare B_4_C	Passive uptake	-	B16-F10	None	First B_4_C nanoparticles for BNCT	No targeting; no in vivo	[53]
NR	NR	Natural/^10^B-B_4_C	Diaminodecane + Brij30 + TAT peptide	TAT peptide	FITC/Lissamine	EL4, B16-F10	None	Enhanced intracellular delivery	No animal validation	[54]
NR	NR	^10^B-enriched B_4_C	Bare nanoparticles	Passive	Intrinsic fluorescence	HeLa, U87MG, MCF-7	None	Higher BNCT efficacy than BPA	No targeting	[56]
NR	PEG −27; PEI +29	Natural BCNO	PEG/PEI/PSMA-CR	Polymer-enhanced uptake	Intrinsic fluorescence	ALTS1C1	None	Low-cost fluorescent BNCT platform	No in vivo	[55]
~80	NR	B_4_C	Human IgG	Macrophage-mediated	FITC	RAW264.7, MC38	None	Long-term stable immune-cell carrier	No animal study	[57]
110 ± 10	NR	98.5% ^10^BPO_4_	PAA + anti-EGFR	EGFR receptor	Cy5.5, IVIS	FaDu	FaDu xenograft	Tumor boron ≈ 63 ppm	HNSCC only	[58]
69–73	−52	^10^B-enriched B_4_C	Hyperbranched polyglycerol	EPR-mediated	Thermal imaging	CT26	CT26 mice	Complete BNCT + PTT tumor eradication	High-RES uptake	[59]
48 ± 18	−21 to −24 (F1); −49 to −52 (F2)	Natural B_4_C	Bare nanoparticles	Passive	-	RAW264.7, J774A.1, JAWS II, NIH/3T3, HT29, MC38, T98G	None	Comprehensive physicochemical characterization	No BNCT	[60]
28.2 ± 0.6	−39	99.63% ^10^B-enriched h-BN	Poly(glycerol)	EPR-mediated	Cy5/Cy7 fluorescence	CT26, RAW264.7	CT26 mice	Tumor boron 102 μg/g; complete BNCT	Colon tumor only	[62]
91 ± 5	−40.8	Natural B_4_C	PAA + Gd shell + DiI	Passive	MRI, fluorescence, neutron autoradiography	HeLa	None	MRI-guided theranostic BNCT	No in vivo therapy	[61]
55 ± 13	−36 ± 0.9	Natural B_4_C	PAA + SPION + DiI	Passive	MRI, fluorescence, neutron autoradiography	HeLa	None	Multifunctional MRI-compatible BNCT platform	No in vivo	[65]
48 ± 18	NR	B_4_C	Bare nanoparticles	Macrophage Trojan horse	TEM, flow cytometry	RAW264.7, J774A.1, JAWS II	None	Cell-mediated delivery	No animal study	[63]
~250	~−20	B_4_C	APTES + PEG_2_K + FA	Folate receptor	FITC	HepG2	HepG2 mice	High tumor boron accumulation	No BNCT study	[66]
NR	NR	B_4_C	Antibody conjugation	LDLR/EGFR	Fluorescence, ICP-MS	SCC-25, T98G, PC-3	None	Highest receptor-specific uptake	No animal validation	[68]
55 ± 13	−36 ± 0.9	Natural B_4_C	PAA-coated Fe_3_O_4_–B_4_C composite	Tumor-infiltrating lymphocytes (ACT/Trojan horse)	MRI, fluorescence, confocal, neutron autoradiography	Jurkat, human melanoma TILs, HeLa coculture	None	First immune-cell-mediated BNCT platform preserving T-cell function and tumor homing	No in vivo validation; proof-of-concept only	[67]

**Table 5:** Carbon-based nanomaterials explored for BNCT, summarizing the material characteristics, boron incorporation, and biological performance.

Size (nm)	Zeta (mV)	Boron Source	Surface Chemistry	Targeting Strategy	Imaging	In Vitro	In Vivo	Advantages	Limitations	Ref.
NR	NR	*nido*-Carborane	Nitrene cycloaddition; water-soluble SWCNTs	Passive (EPR-mediated)	None	None	BALB/c mice bearing EMT6 tumors	High tumor retention (48 h); favorable tumor/blood ratio	No in vitro BNCT or active targeting	[69]
~140 ± 5	NR	Boron-doped carbon	PAA-grafted, PEG–folate	Folate receptor-mediated	MRI, fluorescence	HeLa	None	First folate-targeted carbon BNCT nanocarrier	No in vivo validation	[70]
NR	NR	Boron-10	Folic acid functionalization	Folate receptor-mediated	Fluorescence	NFPA, HeLa, αT3-1	None	High boron uptake in FR-positive cells	No in vivo validation	[71]
NR	NR	BSH	PAMAM dendrimers	Passive	NIR-II fluorescence	None	None	First CNT-based theranostic platform	No biological validation	[72]
~9–10	−2.7 ± 0.3	Boron-doped carbon dots	Tetrazine–PEG	Bioorthogonal pre-targeting (TCO–trastuzumab)	PET/CT, Fluorescence	BT-474	BT-474 xenograft	Ultra-small pre-targeted theranostic platform	No BNCT efficacy study	[73]
~4.5	−41.26	^10^B-boric acid	Boric acid/glucose-derived CQDs	Passive (EPR-mediated)	Intrinsic fluorescence	4T1	BALB/c 4T1 mice	Highest boron loading among carbon dots	Rapid systemic clearance	[74]

**Table 6:** Silicon-based boron nanocarriers for BNCT, emphasizing material design, targeting approaches, and preclinical findings.

Size (nm)	Zeta (mV)	Boron Source	Surface Chemistry	Targeting Strategy	Imaging	In Vitro	In Vivo	Advantages	Limitations	Ref.
NR	~0 (neutral)	o-Carborane (~50 wt%)	Amino-MSN, hydrophobic pores, trivalent galactose, Cy3	ASGPR-mediated active targeting	Cy3 fluorescence	HepG2	None	Very high boron loading; 40–50× higher uptake than BSH	No in vivo validation	[76]
70–76 nm	NR	o-Carborane	ATRP-grown polymer brushes	None	None	None	None	High boron loading (13–18 wt%)	No biological evaluation	[77]
257–427 nm	NR	*closo*-Decaborate	FITC-doped silica	Passive	Fluorescence	None	None	First fluorescent silica BNCT probe	No biological study	[78]
~200 nm	<−25 mV	BSH	PEG/Gd-DTPA/ACPP	MMP-responsive	MRI + fluorescence	CH-2879	Xenograft	MRI-guided BNCT	Moderate boron loading	[79]
170 nm	−42.5 mV	BPA	Phosphonate-functionalized organosilica	LAT1-mediated	Rhodamine	FaDu, A549	CAM model	Biodegradable BPA delivery	No active ligand	[80]
~140–170 nm	~−30 mV	BSH	Organosilica	Passive	Fluorescence	U87MG	Mouse	Improved intracellular BSH delivery	No active targeting	[81]
148–150 nm	~−45 mV	Boric acid	Lipid-coated MSN + SP94 peptide	Active targeting	IVIS fluorescence	HepG2	Xenograft	Tumor boron ~40 ppm	Liver cancer only	[82]

**Table 7:** Liposomal formulations developed for boron delivery in BNCT and their structural features, targeting approaches, and preclinical performance.

DLS (nm)	Zeta (mV)	Boron Source	Targeting Strategy	Imaging	In Vitro	In Vivo	Advantages	Limitations	Ref.
100–150	NR	BSH, Na_3_(B_20_H_17_NH_3_), boronated polyamines	Folate receptor	None	KB	None	Receptor-specific uptake	No in vivo study	[83]
82–96	NR	Carboranyl cholesterol	FR/VEGFR-2	Fluorescence microscopy	KB, 293/KDR	None	Dual-receptor targeting	No therapeutic evaluation	[84]
101–148	+17.5 to +26.4	BPA	HIV-TAT CPP	Fluorescence (tryptophan probe)	None	None	CPP-mediated delivery	Proof-of-concept only	[86]
95–105	−42.8 to −46.7	DSBL/DPBL	Passive	Fluorescence microscopy (PKH67)	Colon-26	BALB/c Colon-26	High in vivo efficacy	High liver uptake	[87]
61/83	NR	MAC + TAC	Passive	None	None	Hamster oral SCC	Excellent biodistribution	No BNCT treatment	[88]
109–134	−76.4	MAC + TAC	Passive	None	EMT6	BALB/c EMT6	High tumor retention	Passive targeting	[90]
111.5	NR	Boron cholesteryl ester	LDL-mediated uptake	None	PC-3	None	Strong therapeutic efficacy	No active targeting	[89]
83	NR	MAC + TAC	Passive	None	None	Hamster oral cancer	Prostate cancer application	In vitro only	[91]
48–200	NR	Polyborane	Passive/PEGylation	None	None	B16 melanoma ddY mice	Size optimization	Biodistribution only	[92]
NR	NR	Arylboronate ester	None	None	None	None	Membrane boron loading	No biological study	[96]
40–42	−10.1 to −5.3	Polyborane	Passive/PEGylation	None	None	B16 melanoma ddY mice	Excellent tumor accumulation	No therapy study	[94]
123.9–124.5	−36.2 to −46.0	BSH	Integrin αvβ3	Fluorescence microscopy	U87, HUVEC	None	Dual-targeted delivery	No animal validation	[93]
48–109	NR	Polyborane	Passive	None	None	B16 melanoma ddY mice	High encapsulation efficiency	No BNCT evaluation	[95]
133.6–143.9	−4.36 to −6.95	BPA-f/B-381	Hyperthermia-triggered	None	D54 glioma	Nude mice	Heat-triggered release	Low encapsulation	[97]
75.3–98.4	−2.4 to −26.1	*o*-Carborane	Passive	None	None	None	Excellent stability	No biological evaluation	[98]
99.2–105.1	−0.72 to −0.20	Polyborane	Passive/PEGylation	None	B16, RAW264.7	B16 ddY mice	Reduced PEG requirement	No therapy study	[99]
~114.5	NR	Potassium nido-carborane	Passive	Fluorescence imaging	CT26	BALB/c CT26	Potent antitumor effect	Passive targeting	[100]
97.9	−18.9	^10^B-BSH	Passive	Fluorescence imaging (Nile Red)	U87, SW620, SK-MEL-28	Orthotopic U87 SCID	Fluorescence tracking	No therapeutic study	[101]
173.8 ± 25.2	−46.5 ± 7.6	BSH-PEG lipid	Passive	None	None	None	Surface boron display	Physicochemical only	[102]
110–120	NR	*nido*-Carborane	Passive	None	CT26	BALB/c CT26	High boron delivery	No added Gd benefit	[103]

**Table 8:** Polymeric and micellar boron delivery systems reported for BNCT, with an emphasis on carrier design, biological evaluation, and therapeutic performance.

Size (nm)	Zeta (mV)	Boron Source	Surface Chemistry	Targeting Strategy	Imaging	In Vitro	In Vivo	Advantages	Limitations	Ref.
~72	NR	Poly(3-acrylamido phenyl boronic acid)	Dextran–boronic acid polymer	None	None	MKN-28, HeLa	None	First boronic acid polymer nanoparticles; tunable size; good stability	No in vivo BNCT; no targeting	[104]
67.3	+0.32	VB-carborane	Covalently polymerized PEG-b-PLA micelles	Passive (EPR)	None	Colon-26	Colon-26 BALB/c mice	Stable boron retention; prolonged circulation; effective BNCT	No active targeting; no imaging	[105]
118.1	NR	Carborane	PEG-polycarbonate with covalent carborane	Passive (EPR)	DOX fluorescence	A549, HeLa	U14 tumor-bearing KM mice	BNCT + chemotherapy; high boron loading; pH-responsive drug release	No active targeting	[106]
46.5, 91.2, 150.1	~0	Carborane	PEG-b-poly(carborane carbonate)	Passive (EPR)	NIR fluorescence	HeLa, A549, L929	U14 tumor-bearing mice	Tunable size; biodegradable; minimal boron leakage	No active targeting	[107]
24.97 ± 3.66	−15.5 ± 1.7	^10^B-BSH	PEG-PCCL polymer + iRGD	iRGD integrin targeting	DOX/ICG fluorescence	A549, B16F10, C6, HeLa, 4T1	A549 & B16F10 xenografts	Excellent biodistribution; very high tumor:blood ratio (14.11); combination therapy	No neutron therapeutic study	[109]
135	NR	Carborane	PEG-b-PLA-galactose polymer	ASGPR (galactose)	Rhodamine fluorescence	HepG2, CAL27, U251	Orthotopic H22 liver tumor-bearing mice	Tumor/blood > 25; excellent HCC targeting; low toxicity	HCC-specific; fluorescence only	[110]
110.1 ± 10.9	−22.4 ± 0.6	o-Carborane	PEGylated covalent organic polymer	Passive (EPR)	PET (^64^Cu) + fluorescence	4T1	4T1 BALB/c mice	High boron loading (11.38 wt%); PET-guided BNCT	No active targeting	[111]
62 ± 5/98 ± 9	NR	Phenylboronic acid	PEG-PLA with terminal PBA	Sialic acid targeting	Fluorescence; CR-39	B16-F10, MDA-MB-231	B16 melanoma mice	Effective BNCT using natural-abundance boron; active targeting	Melanoma only	[113]
150–165	−31 to −37	o-Carborane	PLGA-b-PEG + ACUPA + DFB	PSMA targeting	PET (^89^Zr)	PC3-Pip, PC3-Flu	Dual prostate xenografts	Theranostic platform; FDA-approved polymer	Rapid boron leakage; low tumor boron	[114]
129.9 ± 16.1	-	^10^B-enriched boric acid	PVA crosslinked with boric acid	Passive (EPR); sialic acid interaction	FITC fluorescence	SAS, ALTS1C1	SAS xenograft (SCID mice)	High boron uptake; >24 h circulation; improved BNCT efficacy.	Low tumor-to-blood ratio; no active targeting	[115]
~67	-	Boronate ester polymer	PEG-b-PVBE	Passive (EPR)	Coumarin-6 fluorescence	B16-F10	B16 melanoma mice	38× higher boron uptake; T/B = 2.5; delayed tumor growth	No active targeting	[116]

**Table 9:** Nanoconjugate nanocarriers for BNCT, highlighting boron loading strategies, functionalization, and therapeutic efficacy.

Size (nm)	Zeta (mV)	Boron Source	Composition	Targeting Strategy	Imaging	In Vitro	In Vivo	Advantages	Limitations	Ref.
PVA (Mn ≈ 9.5 kDa)	NR	BPA	Poly(vinyl alcohol) forms reversible boronate ester with BPA	LAT1-mediated endocytosis and prolonged intracellular retention	ICP-MS, confocal microscopy	CT26, BxPC-3	BALB/c, BALB/c nude mice	Increased cellular uptake (≈2.9×), intracellular retention (≈3.6×), prolonged tumor retention, significantly enhanced BNCT efficacy compared with BPA–fructose	No active targeting ligand; no brain tumor model	[117]
6.6 ± 1.1	−8.3 ± 1.1	^10^B-BPA	Poly(β-L-malic acid) conjugated with BPA, angiopep-2, and fluorescent dyes	BBB targeting (angiopep-2) + LAT1-mediated BPA uptake	ICG near-infrared fluorescence, Rhodamine	U87MG	Orthotopic glioblastoma nude mice	Tumor boron 62.7 ± 18.2 ppm, healthy brain 3.8 ± 1.0 ppm, tumor/brain ratio 16.2, excellent BBB penetration	BNCT therapeutic efficacy not evaluated; single glioblastoma model	[17]
NR	NR	BSH	PHPMA polymer conjugated with BSH through a photocleavable linker	Passive EPR accumulation followed by light-triggered blood clearance	None	CT26, NIH3T3	CT26 BALB/c mice	Maintained high tumor boron while reducing blood boron after light exposure; T/B ratio increased from ~1 to ~3; significantly enhanced BNCT efficacy	Requires external 365 nm light; limited penetration depth; only subcutaneous model	[118]

**Table 10:** Dendrimer-based boron carriers for BNCT, summarizing molecular design, targeting strategies, and preclinical outcomes.

Size (nm)	Boron Source	Surface Chemistry	Targeting Strategy	In Vitro	In Vivo	Advantages	Limitations	Ref.
~4	Decaborate clusters (~120–150 B atoms)	PEGylation with folic acid	Folate receptor (FR) targeting	KB cells	FR-positive 24JK-FBP tumor-bearing mice	Active tumor targeting; PEG reduced RES uptake; improved tumor accumulation	High liver/kidney uptake; no BNCT efficacy study	[119]
~5–6	Decaborate clusters (~1100 B atoms)	Site-specific cetuximab conjugation	EGFR/EGFRvIII targeting	F98-EGFR, F98-EGFRvIII glioma	Intracranial F98 glioma rats	Extremely high boron loading; preserved antibody affinity; high tumor boron concentration	Intratumoral administration; no imaging; limited systemic evaluation	[120]
-	Trivalent carborane (~30 B atoms)	Trivalent galactose dendron (CuAAC)	ASGPR-mediated hepatoma targeting	HepG2, HeLa	-	~20-fold higher boron uptake than BSH; >10^10^ B atoms/cell; ~10-fold greater BNCT efficacy than BSH	In vitro only, no biodistribution; no imaging	[121]
-	3 or 9 closo-/nido-carborane clusters	Hydrosilylation; water-soluble nido-carboranes	-	-	-	Very high boron loading; controllable dendrimer generation; fluorescent tracking; water-soluble analogs	No biological validation; no targeting ligand; no BNCT efficacy	[122]

**Table 11:** Comparative characteristics of nanocarriers used in BNCT.

Nanocarrier	Boron Compounds	Boron Loading	Targeting Strategy	Key Advantages	Limitations
Gold NPs	Carborane, COSAN, BPA, B-clusters	Variable; surface-conjugated clusters	Folate, anti-EGFR Ab, TCO–trastuzumab, peptides, radiolabeling (^64^Cu, ^123^I)	Biocompatible; easy surface modification; radiosensitizing; theranostic (MRI, CT, PET, PTT); strong tumor accumulation	High cost; potential long-term toxicity; limited boron density vs. boron NPs
Iron NPs	Carborane (click chemistry), h-BN-Fe_3_O_4_, Boron/Fe_3_O_4_, iron borate	Moderate; surface-attached or doped	External magnetic field guidance; tumor-homing; GSH/H_2_O_2_-responsive (RBNC)	Magnetic-guided delivery; MRI contrast agent; dual MRI + BNCT theranostic; ROS-mediated CDT/ferroptosis synergy	Aggregation under field; complex synthesis; biodistribution via liver/spleen
Boron NPs	Boron carbide (B_4_C), BCNO, h-BN, hexagonal BN (h-^10^BN-PG)	High intrinsic boron density; ~48 µg B/g cell (PEI@BCNO); ^10^B-enriched variants	*Anti*-EGFR Ab; folate receptor (FA); IgG nanoconjugates; TILs as biological carriers; LDLR/EGFR targeting	Extremely high boron content per particle; minimal surface functionalization needed; cell imaging (blue fluorescence); combine PTT + BNCT	Poor aqueous dispersibility; aggregation; scale-up challenges; non-enriched variants less effective
Carbon NPs	Carborane-SWCNTs, BSH-PAMAM/SWCNT, ^10^B-doped C-dots (BAQDs), boron nitride nanographene (BNNG)	Up to 15 wt% ^10^B (BAQDs); surface-functionalized	Folate receptor; HER2 (TCO–trastuzumab pre-targeting); tumor-selective uptake via EPR	Large surface area; high boron loading; NIR-I/II fluorescence imaging; photothermal + chemo + BNCT triple combination; scalable production	Rapid clearance; low passive tumor uptake without targeting; CNT cytotoxicity concerns
Silicon NPs	*o*-Carborane, BSH, BPA (^10^BPA-BPMO), boric acid	Up to ~60 wt% boron; BSH-BPMO with thiol–ene grafting; stable without premature release	Galactose (T-Gal); ACPP (activatable cell-penetrating peptides, Gd); SP94 peptide (liver cancer)	Tunable porosity; high boron loading; perinuclear localization; biodegradable (BPMO); MRI contrast (Gd-MSN); effective in radio-resistant CSCs	Silica non-degradability (standard MSN); moderate in vivo validation; complex synthesis
Liposomes	BSH, *nido*-carborane, polyborane, carboranyl cholesterol, DSBL, PBL, borocaptate (BCH)	High; >100 µg B/g tumor (PEGylated, 12 h post-injection); 22.7 ppm in vivo (DSBL-25% PEG)	Folate receptor; c(RGDyC) for GBM; antibody-modified; thermosensitive (TSL, hyperthermia-triggered); dual-targeting	Excellent biocompatibility; encapsulate hydrophilic & hydrophobic boron; PEGylation extends circulation; high tumor-to-blood ratio; PARP inhibitor co-delivery	Boron leakage over time; lipid bilayer stability challenges; complex multi-step preparation
Polymeric NPs & Micelles	Carborane-PLMB, carborane-polycarbonate, PBA, *m*PEG-b-PBE36, galactose-carborane micelles	38-fold higher than BPA (mPEG-b-PBE36); ~70× above therapeutic threshold (PVA/BA NPs)	iRGD/NRP-1; sialic acid (PBA-NPs); ASGP-R (galactose micelles); PSMA (ACUPA ligands); EPR passive targeting	Structural flexibility; controlled/pH-responsive release; co-delivery with chemo (DOX); size-tunable (EPR optimization); surface PEG reduces clearance	Burst release risk; serum instability; limited boron density vs. inorganic NPs
Nanoconjugates	PVA-BPA boronate ester; PMLA-BPA (Ap2); PHPMA-BSH (photolabile linker)	Covalent attachment; no burst release; precise stoichiometric control	LAT1-mediated (BPA); Ap2 peptide (BBB penetration, GBM); light-controlled photolabile linker for temporal selectivity	Covalent boron conjugation prevents premature release; high tumor-to-blood ratio via light activation; LAT1-targeted efflux inhibition; BBB crossing	Limited number of studies; light-activation requires tumor accessibility; complex chemical synthesis
Dendrimers	PAMAM-borane, boronated dendritic entities (BDE), galactosyl carborane (DGB10), carborane-triphenylbenzene star	Very high; >1100 boron atoms/cetuximab molecule (G5-B1100); ~10× greater cell killing vs. BSH (DGB10)	Anti-EGFR (cetuximab-G5); folate receptor (FA-BDE); ASGP-R (galactosyl DGB10); PEGylation for RES avoidance	Highest boron loading per molecule; precise molecular architecture; monodisperse; fluorescent imaging capability; receptor-mediated selectivity	High liver/kidney accumulation; synthesis complexity per generation; limited in vivo data

## Data Availability

No new data were created or analyzed in this study. Data sharing is not applicable to this article.
